# UHPLC‐DAD‐HRMS^2^ Characterization of Novel Dihydrochalcone Conjugates From the Antiviral *Mansoa hirsuta* Extracts: Diagnostic Fragmentation Pathways and Dereplication of Oligomeric Polyphenols

**DOI:** 10.1002/jms.70128

**Published:** 2026-11-01

**Authors:** Adriana Cotta Cardoso Reis, Breno de Mello Silva, Cíntia Lopes de Brito Magalhães, Erna G. Kroon, Douglas da Costa Gontijo, Markus Kohlhoff, Geraldo Célio Brandão

**Affiliations:** ^1^ Programa de Pós‐graduação em Ciências Farmacêuticas, Escola de Farmácia Universidade Federal de Ouro Preto Ouro Preto Minas Gerais Brazil; ^2^ Departamento de Ciências Biológicas, ICEB Universidade Federal de Ouro Preto Ouro Preto Minas Gerais Brazil; ^3^ Instituto de Ciências Biológicos Universidade Federal de Minas Gerais Belo Horizonte Minas Gerais Brazil; ^4^ Divisão de Química Orgânica, Instituto de Química Universidade de Brasília Brasília Distrito Federal Brazil; ^5^ Laboratório de Química de Produtos Naturais Bioativos, Instituto René Rachou Fundação Oswaldo Cruz Belo Horizonte Minas Gerais Brazil

**Keywords:** antiviral activity, dereplication, flavonoid conjugates, high‐resolution mass spectrometry, liquid chromatography

## Abstract

High‐resolution mass spectrometry (HRMS) coupled with ultra‐high‐performance liquid chromatography (UHPLC) enables rapid characterization of complex polyphenolic constituents in medicinal plants. *Mansoa hirsuta* (Bignoniaceae), a neotropical liana traditionally used to treat infectious diseases, remains poorly characterized chemically. This study reports the comprehensive UHPLC–DAD–HRMS/MS profiling of ethanolic extracts from leaves, stems, and fruits, leading to the identification of 57 phenolic compounds, including 40 previously unreported flavonoid conjugates. Accurate mass measurements and diagnostic MS/MS fragmentation patterns enabled the structural characterization of five classes of dihydrochalcone‐derived metabolites: dihydrochalcone‐tyramine, ‐flavanone (mansoin‐type), ‐flavan‐3‐ol, and ‐flavonol conjugates. Characteristic fragment ions, particularly those corresponding to the dihydrochalcone core and tyramine moiety, supported the annotation of novel oligomeric flavonoids. Organ‐specific metabolite distribution revealed leaves enriched in Mansoin A–type heterotrimers, stems containing phenylethanoid glycosides and cinnamoyl‐mangiferins, and fruits predominated by simpler dihydrochalcone‐tyramine conjugates. All extracts exhibited low cytotoxicity (CC_50_ > 400 μg mL^−1^) and selective antiviral activity against Zika (ZIKV), chikungunya (CHIKV), Mayaro (MAYV), and dengue virus serotype 2 (DENV‐2). The stem extract showed the highest selectivity against ZIKV (SI > 25.5), whereas the fruit extract displayed the greatest potency against DENV‐2 (EC_50_ = 25.4 ± 1.2 μg mL^−1^; SI > 19.7). These findings expand the phytochemical knowledge of *M. hirsuta* , demonstrate the utility of HRMS‐based metabolite profiling for dereplication of complex flavonoids, and provide diagnostic MS/MS fragmentation patterns applicable to the characterization of related oligomeric flavonoids in other plant species.

## 1 Introduction

Liquid chromatography high‐resolution mass spectrometry (LC–HRMS) has emerged as one of the most powerful analytical platforms for characterizing complex natural product mixtures. The combination of ultra‐high‐performance liquid chromatography (UHPLC) with high‐resolution tandem mass spectrometry enables rapid separation, accurate mass measurement, and structurally informative fragmentation analysis of metabolites directly from crude plant extracts [1, 2, 3, 4]. Accurate mass measurements (typically ≤ 5 ppm), isotopic pattern analysis, and MS/MS fragmentation data support confident elemental composition assignment and facilitate dereplication, structural annotation, and discovery of previously unreported metabolites in complex matrices [3, 4, 5]. Furthermore, the integration of diode‐array detection (DAD) with HRMS provides complementary spectroscopic information essential for natural products, mainly phenolic compounds. UV absorption patterns assist in recognizing characteristic chromophores of flavonoids and related polyphenols, whereas high‐resolution MS and MS/MS experiments reveal diagnostic fragmentation pathways that enable classification of conjugated flavonoid structures.

The genus *Mansoa* (Bignoniaceae) comprises neotropical lianas widely distributed in South America with traditional medicinal use. *Mansoa hirsuta* D.C., popularly known as “cipó‐de‐alho,” occurs in Brazilian biomes including Cerrado, Caatinga, and Atlantic Forest. Ethnopharmacological reports describe its use against infections, inflammatory disorders, and gastrointestinal conditions [6, 7]. Despite this relevance, the chemical composition of *M. hirsuta* remains only partially explored. Previous studies led to the isolation of six unusual heterotrimeric flavonoids, mansoin A–F, representing structurally complex polyphenolic constituents [8, 9].

The chemical diversity present in plant extracts offers a valuable reservoir of structurally novel scaffolds that may complement existing antiviral strategies. Indeed, natural products constitute an important source of antiviral leads, and several classes of plant secondary metabolites, including flavonoids and phenylethanoid glycosides, have demonstrated inhibitory activity against RNA viruses through diverse mechanisms [10, 11, 12, 13].

Thus, the present study aimed to (i) perform comprehensive chemical characterization of these extracts using UHPLC coupled to DAD and high‐resolution tandem mass spectrometry and (ii) evaluate the antiviral effects of ethanolic extracts (EEs) from leaves, stems, and fruits of *M. hirsuta* against dengue virus (DENV), Zika virus (ZIKV), Mayaro virus (MAYV), and chikungunya virus (CHIKV), viruses that represent significant public health burdens in tropical and subtropical regions, with limited therapeutic options.

## 2 Materials and Methods

### 2.1 Plant Material and Extract Preparation

*Mansoa hirsuta* D.C. samples were collected at the Estação Biológica de Caratinga conservation unit, Minas Gerais, Brazil. Botanical identification was performed by Dr. J. A. Lombardi (Department of Botany, Institute of Biosciences, UNESP, Rio Claro, Brazil), and a voucher specimen (BHCB 23862) was deposited in the BHCB/UFMG Herbarium, Belo Horizonte, Brazil.

Fresh plant material was separated into stems, leaves, and fruits, washed, and dried in a ventilated oven at 40°C for 72 h. Dried materials were milled to homogeneous powder and exhaustively extracted by room‐temperature percolation with absolute ethanol for 48 h. The extracts were filtered and concentrated under reduced pressure at 50°C using a rotary evaporator to yield crude EEs, which were stored at −20°C until analysis. Extractive yields were 19.2 g (leaves), 11.6 g (stems), and 10.8 g (fruits) from 62.4, 56.5, and 28.7 g of dried material, respectively.

### 2.2 UHPLC–HRMS/MS Analysis

Chemical profiling was performed using a Nexera UHPLC system (Shimadzu, Kyoto, Japan) coupled to a maXis electrospray ionization quadrupole time‐of‐flight mass spectrometer (ESI‐QTOF‐MS; Bruker Daltonics, Bremen, Germany) operated in high‐resolution mode [14]. Chromatographic separation was achieved on a Shim‐Pack XR‐ODS‐III C18 column (2.0 × 150 mm, 2.2‐μm particle size) maintained at 40°C. The mobile phase consisted of water with 0.1% formic acid (A) and acetonitrile with 0.1% formic acid (B). A linear gradient from 5% to 100% B over 40 min was applied, followed by a 5‐min hold at 100% B. The flow rate was 0.40 mL min^−1^, and the injection volume was 5 μL of extract solution (1 mg mL^−1^ in methanol) [14]. After UV‐PDA detection (190–450 nm), mass spectra were acquired in positive mode at a rate of 5 Hz. Ion‐source parameters were set to 500‐V end‐plate offset, 4500‐V capillary voltage, 3.0‐bar nebulizer pressure, and 8 L min^−1^ and 200°C dry gas flow and temperature, respectively. Data‐dependent fragment spectra were recorded using a collision energy range between 15 and 60 eV. Ion cooler settings were optimized for an *m/z* range of 100–1500. External calibration was performed using sodium formate, and post‐acquisition internal recalibration was applied to maintain mass accuracy. Data‐dependent MS/MS experiments were conducted using collision‐induced dissociation with stepped collision energies. Molecular formula assignment and putative compound identification were based on accurate mass measurements (≤ 5 ppm error), isotopic pattern analysis, retention time, and comparison of MS/MS fragmentation with literature data [4, 5]. Mass spectral data were processed using Bruker Compass DataAnalysis software. Relative peak area percentages were calculated from extracted ion chromatogram (EIC) peak areas and normalized to the total summed area of all phenolic compounds detected in each respective extract.

### 2.3 Cell Culture

Vero cells (African green monkey kidney, ATCC CCL‐81) LLC‐MK2 cells (rhesus monkey kidney, ATCC CCL‐7), and MRC‐5 cells (human lung fibroblast, ATCC CCL‐171) were cultured in Dulbecco's modified Eagle's medium (DMEM) supplemented with 5% fetal bovine serum (FBS), 100‐U mL^−1^ penicillin/streptomycin, and 5‐μg mL^−1^ amphotericin B. Cells were maintained at 37°C in a humidified 5% CO_2_ atmosphere. Zika virus (ZIKV), chikungunya virus (CHIKV), and Mayaro virus (MAYV) were obtained from collaborating laboratories. Viral titers were determined as 6.8 × 10^5^ PFU mL^−1^ (ZIKV), 1.44 × 10^11^ PFU mL^−1^ (CHIKV), and 2.5 × 10^8^ PFU mL^−1^ (MAYV) [15, 16].

### 2.4 Cytotoxicity Assay

Extracts were dissolved in dimethyl sulfoxide (DMSO) to obtain stock solutions (10 mg mL^−1^). Cell monolayers were seeded in 96‐well plates at 2.0 × 10^4^ cells per well and incubated for 24 h. Serial dilutions of extracts (400–0.78 μg mL^−1^) were prepared in DMEM containing 1% FBS and added to the wells. After incubation (48 h for MRC‐5 and 72 h for Vero and LLC‐MK2 cells), cell viability was evaluated using the MTT colorimetric assay [17]. Formazan crystals were dissolved in DMSO, and absorbance was measured at 492 nm using a multiwell spectrophotometer (Victor X3, PerkinElmer). The 50% cytotoxic concentration (CC_50_) was calculated from concentration–response curves using GraphPad Prism software. Experiments were performed in quadruplicate.

### 2.5 Antiviral Assays

Antiviral activity was evaluated in Vero cells using an MTT‐based cytopathic effect inhibition assay. Cell monolayers were infected with viral suspensions at predetermined titers (1.0 × 10^3^ TCID_50_ mL^−1^ for ZIKV, 1.0 × 10^7^ TCID_50_ mL^−1^ for CHIKV, and 1.0 × 10^5^ TCID_50_ mL^−1^ for MAYV) and simultaneously treated with non‐cytotoxic extract concentrations (100–0.78 μg mL^−1^). Plates were incubated for 72 h at 37°C in 5% CO_2_ [15, 16, 18].

Controls included untreated infected cells, treated uninfected cells, and untreated uninfected cells. Ribavirin, amantadine, and α‐2a interferon were used as positive antiviral controls. The effective concentration required to inhibit 50% of virus‐induced cytopathic effects (EC_50_) was determined from concentration–response curves. The selectivity index (SI) was calculated as CC_50_/EC_50_ [19].

### 2.6 Statistical Analysis

Data are expressed as mean ± standard deviation of at least three independent experiments. CC_50_ and EC_50_ values were obtained by nonlinear regression analysis using GraphPad Prism 5.0 software. Statistical comparisons were performed using Student's *t*‐test, with *p* < 0.05 considered statistically significant.

## 3 Results and Discussion

### 3.1 Phytochemical Overview

A total of 57 phenolic compounds were comprehensively characterized across the leaves, stem, and fruit ethanol extracts from *M. hirsuta* . Phenolic compounds, including flavonoids, xanthones, and phenylethanoids, were putatively identified by their characteristic UV absorptions at 254, 280, and 320 nm, consistent with established chromatographic behavior of polyphenolic structures [20], and mass spectrometric diagnostic fragmentations. The detailed phytochemical profile of the three extracts is presented in Table 1. Thus, the structural diversity observed reflects the complex biosynthetic capacity of *M. hirsuta* , with metabolites organized into five distinct structural groups based on their central flavonoid scaffolds: (i) dihydrochalcone‐tyramine conjugates, (ii) dihydrochalcone‐flavanone conjugates (Mansoin type), (iii) dihydrochalcone‐flavan‐3‐ol conjugates, (iv) dihydrochalcone‐flavone conjugates, and (v) dihydrochalcone‐flavonol conjugates.

**TABLE 1:** UHPLC‐DAD‐HRMS^2^ putative identification and percentages of relative area of the peaks from the putative identified compounds in the leaves, stems, and fruits ethanolic extracts of the *Mansoa hirsuta*.

	Compound	Molecular formula	HRMS *m/z* [M + H]^+^	Δ ppm	Characteristic *m/z* of ions in positive ion mode (%)	UV	RT (min)	EE leaves %	EE stems%	EE fruits%
**1**	2′,4′,4‐Trihydroxydihydrochalcone	C_15_H_14_O_4_	259.0967	1.1	259.0969 (100.0), 197.1179 (10.4), 153.0544 (81.8)	219, 276 (sh)	14.3	—	—	3.44
**2**	Eriodictyol	C_15_H_12_O_6_	289.0708	1.4	289.0710 (100.0), 163.0393 (28.6), 153.0183 (74.7), 135.0435 (11.9)	219, 276 (sh)	15.3	—	—	2.8
**3**	Flavonoid conjugates containing dihydrochalcone and tyramine moieties	C_23_H_25_NO_4_	380.1861	0	380.1864 (16.3), 243.1018 (100.0), 138.0915 (13.5), 137.0597 (14.1), 123.0441 (6.1), 121.0645 (12.8)	219	12.5	—	—	2.91
**4**	Apigenin‐*O*‐glucuronide	C_21_H_18_O_11_	447.0919	1.8	447.0913 (28.5), 271.0602 (100), 153.0181 (4.1)	197, 220, 274	15.7	2.98	—	—
**5**	Kaempferol‐*O*‐glucoside	C_21_H_20_O_11_	449.1079	2.2	449.1075 (3.0), 287.0552 (100.0), 153.0179 (2.3)	274, 352	15.1	13.1	—	—
**6**	6‐Hidroxyluteolin‐*O*‐glucoside	C_21_H_20_O_12_	465.1023	2.1	465.1014 (3.4), 303.0497 (100.0), 153.0184 (3.8)	218	14.0	1.49	—	—
**7**	Flavonoid conjugates containing dihydrochalcone and tyramine moieties	C_29_H_35_NO_8_	526.2438	0.4	526.2424 (47.4), 243.1016 (100.0), 149.0568 (3.4), 137.0596 (16.1), 133.0647 (12.1), 123.0439 (5.1)	219	13.1	—	—	0.58
**8**	Flavonoid conjugates containing dihydrochalcone and tyramine moieties	C_29_H_35_NO_8_	526.2439	0.2	526.2435 (53.1), 309.1128 (2.3), 243.1017 (100.0), 137.0594 (12.2), 133.0643 (14.4), 123.0435 (8.4)	219	13.7	—	—	3.37
**9**	Flavonoid conjugates containing dihydrochalcone and tyramine moieties	C_29_H_35_NO_9_	542.2395	0.9	542.2392 (68.9), 405.1548 (1.7), 243.1020 (100.0), 137.0596 (11.8), 133.0647 (15.2), 123.0440 (5.2)	196, 220, 276	11.5	—	9.94	20.48
**10**	Flavonoid conjugates containing dihydrochalcone and tyramine moieties	C_29_H_35_NO_9_	542.2394	0.7	542.2391 (68.3), 405.1548 (1.7), 243.1020 (100.0), 137.0596 (12.4), 133.0647 (15.7), 123.0439 (5.4)	196, 220, 276	11.6	—	1.36	2.31
**11**	Flavonoid conjugates containing dihydrochalcone and tyramine moieties	C_29_H_35_NO_9_	542.2395	0.9	542.2390 (67.4), 405.1549 (1.7), 243.1020 (100.0), 137.0596 (13.4), 133.0646 (16.7), 123.0439 (5.8)	196, 220, 276	11.9	—	11.77	32.54
**12**	Flavonoid conjugates containing dihydrochalcone and tyramine moieties	C_29_H_35_NO_9_	542.2393	0.5	542.2390 (66.1), 405.1547 (1.7), 243.1020 (100.0), 137.0596 (13.7), 133.0646 (16.8), 123.0439 (6.1)	197, 219, 275	12.2	—	1.09	2.87
**13**	2′‐*O*‐*p*‐hydroxy‐benzoylmangiferin (Muraxanthone)	C_26_H_22_O_13_	543.1113	4.6	543.1110 (80.4), 423.0695 (16.1), 303.0489 (38.6), 285.0385 (100), 283.0805 (14.8), 273.0383 (19.1), 257.0438 (28.2), 121.0277 (40.0)	200, 220, 273, 325	14.4	—	3.19	0
**14**	2′‐*O*‐*trans*‐cinnamoylmangiferin	C_28_H_24_O_12_	553.1318	5.0	553.1313 (97.7), 433.0901 (20.6), 303.0491 (20.2), 293.1009 (15.9), 285.0385 (95.5), 273.0386 (20.7), 257.0436 (28.0), 131.0483 (100.0)	195, 225, 284, 353	18.1	—	4.61	—
**15**	2′‐*O*‐*trans*‐coumaroylmangiferin	C_28_H_24_O_13_	569.1271	4.2	569.1266 (40.7), 327.0492 (7.5), 303.0489 (18.2), 285.0386 (27.5), 273.0384 (11.4), 257.0438 (8.9), 147.0434 (100.0), 119.0484 (6.4)	199, 220, 280, 325	15.3	—	3.35	—
**16**	2′‐*O*‐*trans*‐caffeoylmangiferin	C_28_H_24_O_14_	585.1215	4.9	585.1208 (39.9), 423.0900 (35.4), 327.0500 (15.6), 303.0496 (29.3), 285.0387 (32.7), 273.0391 (20.8), 163.0385 (100.0)	200, 220, 280, 325	14.5	—	0.71	—
**17**	Verbascoside	C_29_H_36_O_15_	625.2103	4.6	479.1527 (3.5), 471.1477 (10.5), 325.0907 (53.1), 309.0958, 181.0491 (3.1), 163.0383 (100.0), 145.0276 (3.6)	199, 326	14.2	—	13.9	—
**18**	Isoverbascoside	C_29_H_36_O_15_	625.2101	4.9	479.1526 (5.6), 477.1359 (5.8), 471.1464 (5.8), 325.0906 (27.8), 309.0947 (4.6), 307.0801 (5.4), 181.0492 (7.1), 163.0384 (100.0)	220, 323	14.7	—	4.07	—
**19**	Flavonoid conjugates containing dihydrochalcone and flavanone moieties	C_36_H_36_O_13_	677.2237	0.4	515.1702 (52.1), 405.1336 (100.0), 303.0867 (12.5), 299.0918 (65.4), 285.0760 (45.8), 243.1021 (33.3), 179.0341 (44.0)	196, 220, 284	19.2	—	—	16.19
**20**	Flavonoid conjugates containing dihydrochalcone and flavan‐3‐ol moieties	C_36_H_38_O_13_	679.2387	0.4	517.1849 (12.4), 407.1486 (100.0), 301.1070 (60.0), 287.0919 (7.8), 243.1007 (8.0), 195.0650 (18.1), 181.0495 (6.7), 133.0645 (8.9)	197, 220, 284	18.3	4.63	—	—
**21**	Flavonoid conjugates containing dihydrochalcone and flavanone moieties	C_36_H_36_O_14_	693.2180	0.4	693.2173, 531.1644 (42.3), 421.1280 (100.0), 315.0867 (66.7), 303.0853 (13.9), 285.0758 (35.9), 243.1016 (57.0), 179.0341 (38.1)	196, 220, 284	18.1	9.57	5.53	9.72
**22**	Flavonoid conjugates containing dihydrochalcone and flavanone moieties	C_42_H_46_O_18_	839.2773	1.3	677.2230 (35.8), 515.1703 (55.3), 405.1338 (94.8), 299.0918 (47.3), 285.0762 (36.1), 243.1020 (100.0)	193, 220, 283	15.9	—	—	16.30
**23**	Flavonoid conjugates containing dihydrochalcone and flavan‐3‐ol moieties	C_42_H_48_O_18_	841.2929	1.3	679.2379 (8.3), 569.2012 (12.0), 517.1851 (16.3), 407.1488 (100.0), 301.1070 (48.1), 243.1017 (21.5), 195.0654 (9.8), 133.0644 (10.1)	197, 220, 284	18.5	4.33	—	7.32
**24**	Flavonoid conjugates containing dihydrochalcone and flavone moieties	C_42_H_44_O_19_	853.2562	0.8	853.2552 (33.8), 691.2017 (30.3), 529.1489 (100.0), 419.1124 (57.4), 313.0707 (31.6), 243.1017 (27.2)	197, 225, 273	16.1	8.01	—	—
**25**	Flavonoid conjugates containing dihydrochalcone and flavone moieties	C_42_H_44_O_19_	853.2563	0.9	853.2553 (32.0), 691.2018 (38.3), 529.1491 (100.0), 419.1124 (56.6), 313.0707 (30.5), 243.1017 (18.8)	197, 225, 273	16.2	1.82	—	—
**26**	Flavonoid conjugates containing dihydrochalcone and flavone moieties	C_42_H_44_O_19_	853.2564	1.0	853.2554 (31.0), 691.2017 (30.3), 529.1489 (100.0), 419.1124 (57.4), 313.0707 (31.6), 243.1017 (27.2)	197, 225, 274	16.4	9.53	—	—
**27**	Flavonoid conjugates containing dihydrochalcone and flavone moieties	C_42_H_44_O_19_	853.2562	0.8	853.2556 (30.5), 691.2024, (43.1), 529.1489 (100.0), 419.1124 (50.6), 313.0707 (30.1), 243.1020 (16.1)	197, 280	16.6	0.79	—	—
**28**	Flavonoid conjugates containing dihydrochalcone and flavanone moieties (*Mansoin C type*)	C_42_H_46_O_19_	855.2720	1.0	693.2169 (27.1), 531.1640 (27.6), 421.1279 (53.2), 315.0863 (35.4), 285.0759 (18.2), 243.1018 (100.0)	197, 280	16.8	7.19	7.62	32.84
**29**	Flavonoid conjugates containing dihydrochalcone and flavanone moieties (*Mansoin C type*)	C_42_H_46_O_19_	855.2717	0.7	693.2165 (13.2), 583.1803, 421.1280 (76.9), 315.0864 (56.5), 289.0707 (13.7), 285.0760 (13.2), 243.1017 (100.0). 179.0340 (12.8)	196, 283	17.6	3.12	3.44	9.44
**30**	Flavonoid conjugates containing dihydrochalcone and flavonol moieties	C_42_H_44_O_20_	869.2508	0.5	869.2497 (41.2), 707.1962 (35.1), 545.1436 (100.0), 435.1070 (56.1), 329.0655 (41.0), 243.1016 (36.8)	199, 220, 276	15.5	8.5	3.26	—
**31**	Flavonoid conjugates containing dihydrochalcone and flavonol moieties	C_42_H_44_O_20_	869.2512	0.9	869.2504 (33.8), 707.1965 (43.3), 545.1439 (100.0), 435.1072 (52.9), 329.0657 (26.8), 243.1018 (22.9)	197, 220, 273	15.9	2.26	1.36	—
**32**	Flavonoid conjugates containing dihydrochalcone and flavanone moieties	C_51_H_50_O_17_	935.3133	0.7	663.2216 (77.6), 553.1860 (46.3), 447.1440 (100.0), 421.1277 (65.7), 417.1333 (39.2), 405.1538 (31.2), 243.1017 (76.1)	197, 284	21.0	—	—	0.77
**33**	Flavonoid conjugates containing dihydrochalcone and tyramine moieties	C_50_H_60_NO_17_	946.3863	0.2	946.3880 (21.3), 809.3015, 647.2497, 485.1956 (42.0), 375.1594 (23.6), 269.1176 (18.0), 243.1018 (100.0), 133.0646 (16.2)	219, 276	13.9	—	—	4.75
**34**	Flavonoid conjugates containing dihydrochalcone and tyramine moieties	C_50_H_58_NO_17_	946.3861	0	946.3860 (23.0), 809.2941, 647.2505, 485.1956 (42.0), 375.1594 (22.0), 349.1437 (13.0), 269.1176 (13.7), 243.1017 (100.0), 133.0645 (21.4)	219, 276	14.4	—	—	2.47
**35**	Flavonoid conjugates containing dihydrochalcone and flavone moieties	C_51_H_50_O_21_	999.2938	1.6	999.2934 (17.7), 691.2013 (14.9), 529.1494 (46.7), 419.1122 (16.5), 309.0973 (26.6), 291.0870 (12.1), 243.1024 (23.6), 147.0438 (100.0)	197, 283	17.3	3.44	—	—
**36**	Flavonoid conjugates containing dihydrochalcone and tyramine moieties	C_51_H_57_NO_20_	1004.3550	0.2	1004.3549 (46.5), 867.2707, 705.2174 (36.1), 595.1809 (100.0), 433.1284 (94.4), 327.0863 (31.3), 297.0760 (23.2), 243.1019	219, 276	14.7	—	—	0.98
**37**	Flavonoid conjugates containing dihydrochalcone, flavanone and tyramine moieties	C_51_H_57_NO_20_	1004.3557	0.5	1004.3550 (46.7), 867.2707, 705.2173 (36.4), 595.1809 (100.0), 489.1395 (22.8), 433.1284 (94.5), 327.0863 (31.5), 297.0759 (23.3), 243.1019	219, 276	14.9	—	—	0.73
**38**	Flavonoid conjugates containing dihydrochalcone and flavone moieties	C_60_H_58_O_18_	1067.3681	1.9	663.2193 (54.2), 557.1815 (35.3), 553.1851 (54.6), 531.1666 (26.5), 447.1423 (68.6), 421.1263 (39.5), 243.1024 (100.0), 133.0635 (26.1)	197, 284	20.7	10.9	—	—
**39**	Flavonoid conjugates containing dihydrochalcone and flavanone moieties (*Mansoin B type*)	C_57_H_60_O_22_	1097.3680	2.4	1097.3663, 935.3123, 825.2751, 693.2170 (9.9), 663.2220 (9.4), 553.1852 (9.1), 531.1645 (8.4), 447.1436 (14.0), 421.1279 (21.1), 405.1541 (19.4), 243.1018 (100.0)	196, 284	19.3	8.89	7.0	16.81
**40**	Flavonoid conjugates containing dihydrochalcone and flavanone moieties (*Mansoin B type*)	C_57_H_60_O_22_	1097.3673	1.7	1097.3679, 935.3135, 825.2749, 693.2184 (8.1), 663.2214 (11.3), 553.1850 (9.7), 447.1430 (16.3), 421.1274 (28.7), 405.1540 (15.7), 243.1015 (100.0), 133.0642 (10.0)	197, 284	19.8	1.90	3.7	5.53
**41**	Flavonoid conjugates containing dihydrochalcone and flavanone moieties (*Mansoin B type*)	C_57_H_60_O_22_	1097.3679	2.3	1097.3673, 935.3124, 825.2748, 693.2171 (10.7), 663.2217 (13.9), 553.1850 (13.2), 531.1642 (10.3), 447.1434 (22.2), 421.1279 (22.4), 405.1540 (15.1), 243.1015 (100.0), 133.0643 (8.8)	196, 285	20.4	41.84	25.76	41.47
**42**	Flavonoid conjugates containing dihydrochalcone and flavanone moieties (*Mansoin B type*)	C_57_H_60_O_22_	1097.3679	2.3	1097.3678, 935.3130, 825.2753 (10.7), 693.2173 (10.3), 663.2222 (18.8), 553.1854 (17.0), 447.1437 (27.7), 421.1280 (24.5), 417.1331 (10.2), 405.1542 (17.7), 243.1018 (100.0)	198, 220, 285	21.1	19.54	14.7	13.08
**43**	Flavonoid conjugates containing dihydrochalcone and flavone moieties	C_63_H_68_O_27_	1257.4069	3.4	1257.4069, 1095.3413, 9 332 954, 771.2426 (28.5), 485.1957 (36.4), 419.1125 (72.1), 313.0704 (93.3), 287.0550 (25.4), 243.1014 (100.0)	197, 273	15.9	2.26	—	—
**44**	Flavonoid conjugates containing dihydrochalcone and flavone moieties	C_63_H_68_O_27_	1257.4067	3.3	1257.4072, 1095.3514, 933.2962 (21.6), 771.2441 (40.2), 485.1957 (33.3), 419.1118 (93.2), 313.0707 (100.0), 287.0553 (28.7), 243.1016 (84.0)	197, 273	16.2	2.33	—	—
**45**	Flavonoid conjugates containing dihydrochalcone and flavone moieties	C_63_H_68_O_27_	1257.4070	3.5	1257.4122, 1095.3506, 9 332 967, 823.2593 (48.0), 691.2018 (27.5), 661.2063 (60.7), 555.1647 (34.1), 529.1496 (25.9), 445.1282 (32.2), 419.1120 (38.1), 243.1018 (100.0)	197, 281	17.3	3.44	—	—
**46**	Flavonoid conjugates containing dihydrochalcone and flavone moieties	C_63_H_68_O_27_	1257.4072	3.6	1257.4126, 1095.3503, 9 332 966, 823.2594 (53.1), 691.2021 (28.5), 661.2063 (67.2), 555.1647 (37.3), 529.1496 (27.2), 529.1496 (34.1), 419.1119 (33.9), 243.1018 (100.0)	197, 281	17.4	2.05	—	—
**47**	Flavonoid conjugates containing dihydrochalcone and flavanone moieties (*Mansoin A type*)	C_63_H_70_O_27_	1259.4157	2.0	1259.4220, 1097.3661, 855.2712, 693.2165 (5.1), 583.1802 (4.4), 567.2069 (4.6), 531.1634 (5.7), 421.1280 (15.8), 405.1541 (12.4), 243.1018 (100.0), 137.0593 (4.7), 133.0642 (8.1)	196, 282	17.5	2.07	4.07	2.7
**48**	Flavonoid conjugates containing dihydrochalcone and flavanone moieties (*Mansoin A type*)	C_63_H_70_O_27_	1259.4224	3.3	1259.4240, 1097.3670, 987.3278, 855.2707, 693.2168 (5.0), 583.1804 (4.3), 567.2065 (5.1), 531.1640 (4.4), 421.1279 (16.2), 405.1541 (13.5), 243.1018 (100.0), 137.0592 (4.4), 133.0644 (8.6)	196, 284	18.1	10.30	—	21.54
**49**	Flavonoid conjugates containing dihydrochalcone and flavanone moieties (*Mansoin A type*)	C_63_H_70_O_27_	1259.4231	3.9	1259.4241, 1097.3680, 987.3295, 855.2707 (4.5), 693.2173 (6.1), 531.1646 (6.0), 421.1281 (15.2), 405.1543 (13.4), 243.1017 (100.0)	197, 284	18.8	48.42	47.73	68.88
**50**	Flavonoid conjugates containing dihydrochalcone and flavanone moieties (*Mansoin A type*)	C_63_H_70_O_27_	1259.4227	3.6	1259.4241, 1097.3673, 987.3285, 855.2705, 693.2171 (5.6), 583.1802 (4.9), 567.2066 (4.9), 531.1644 (5.6), 421.1279 (17.2), 405.1543 (16.7), 243.1017 (100.0), 133.0643 (7.6)	197, 284	19.7	13.93	17.0	24.49
**51**	Flavonoid conjugates containing dihydrochalcone and flavanone moieties (*Mansoin A type*)	C_63_H_70_O_27_	1259.4243	4.8	1259.4243, 1097.3674, 987.3294, 855.2705, 693.2174, 583.1792 (4.7), 567.2062 (4.7), 531.1650 (4.6), 421.1277 (14.5), 405.1541 (16.9), 243.1016 (100.0), 137.0595 (4.3), 133.0643 (8.6)	197, 284	20.0	1.37	—	46.61
**52**	Flavonoid conjugates containing dihydrochalcone and flavonol moieties	C_63_H_68_O_28_	1273.4019	3.4	1273.4090, 1111.3469, 949.2995, 787.2385 (24.3), 485.1963 (27.6), 435.1069 (73.4), 329.0651 (74.4), 303.0496 (21.1), 243.1015 (100.0)	198, 275	15.6	1.39	—	—
**53**	Flavonoid conjugates containing dihydrochalcone and flavonol moieties	C_63_H_68_O_28_	1273.4028	4.2	1273.4090, 1111.3575, 949.2915 (18.7), 787.238 (26.9), 485.1954 (30.4), 435.1072 (86.9), 329.0653 (79.8), 303.0496 (24.3), 243.1018 (100.0)	197, 274	15.7	2.98	—	—
**54**	Flavonoid conjugates containing dihydrochalcone and flavanone moieties	C_72_H_74_O_25_	1339.4651	4.0	1339.4651, 809.3003, 647.2463 (26.4), 553.1883 (25.0), 485.1943 (40.4), 447.1423 (15.7), 421.1279 (224.7), 405.1529 (16.6), 375.1595 (14.1), 243.1020 (100.0), 133.0641 (15.4)	196, 284	19.4	3.07	—	—
**55**	Flavonoid conjugates containing dihydrochalcone and flavanone moieties	C_72_H_74_O_25_	1339.4651	4.0	1339.4672, 663.2217 (25.0), 647.2479 (27.6), 553.1851 (15.0), 485.1954 (49.6), 421.1276 (48.8), 405.1542 (18.1), 375.1589 (15.2), 243.1017 (100.0)	197, 284	20.7	10.90	8.08	—
**56**	Flavonoid conjugates containing dihydrochalcone and flavanone moieties	C_69_H_80_O_32_	1421.4750	2.7	1421.4768, 971.3560, 855.2735, 693.2197, 567.2069 (25.5), 421.1289 (16.8), 405.1543 (41.3), 325.1133 (7.6), 243.1017 (100.0), 145.0498 (7.0), 133.0653 (10.1)	197, 283	17.3	—	—	1.78
**57**	Flavonoid conjugates containing dihydrochalcone and flavanone moieties	C_69_H_80_O_32_	1421.4745	2.4	1421. 4646, 855.2717, 693.2189 (7.1), 567.2080 (28.2), 531.1650 (7.1), 421.1285 (14.8), 405.1546 (31.9), 325.1127 (10.7), 243.1018 (100.0)	197, 284	17.6	—	—	2.92

*Note:* The putative compounds were numbered according to *m/z*, from lowest to highest.

Abbreviations: (%): relative peak area percentages; (—) not detected; EE: ethanolic extracts; RT: retention time; sh: shoulder.

A remarkable feature of the *M. hirsuta* extracts was the prevalence of compounds exhibiting a base peak fragment ion at *m/z* 243.1018 [M + H]^+^. This specific fragment corresponds putatively to the 2′,4′,4‐trihydroxydihydrochalcone residue, a structural motif previously identified as the core unit of Mansoin A type, a heterotrimeric flavonoid isolated from this species [7, 8, 9]. The fragment ions, fragmentation patterns, and connectivity between flavonoid units established for Mansoin A (Figures 1 and S1) served as diagnostic templates for proposing structures of related conjugate flavonoids detected in this study. Another significant structural class was characterized by the neutral loss of 137 Da (C_8_H_11_NO), indicative of tyramine residue incorporation into the flavonoid skeleton [21]. This neutral loss pattern, combined with the characteristic *m/z* 243.1018 [M + H]^+^ dihydrochalcone fragment, enabled the identification of dihydrochalcone‐tyramine conjugates (compounds 3, 7–12, 33–34, 36–37).

**FIGURE 1:**
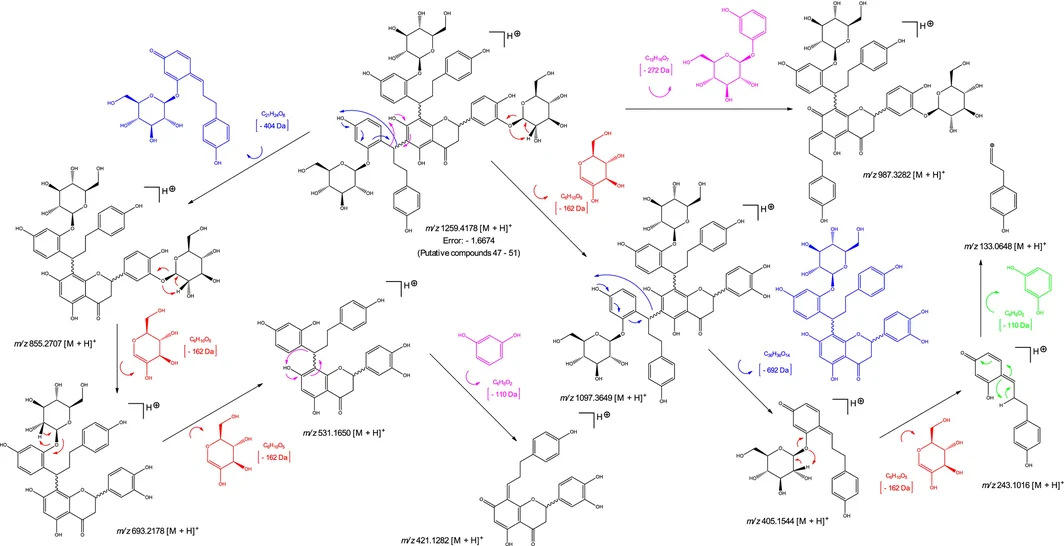
Proposed fragmentation pathways of ion *m/z* 1259.4178 [M + H]^+^, putatively identified as isomers of the conjugate dihydrochalcone and flavanone moieties (Mansoin A Type), detected in the *M. hirsuta* extracts. Putative isomers of compounds 47, 48, 49, 50, and 51.

The phytochemical composition varied significantly among plant organs, reflecting tissue‐specific biosynthesis and accumulation: (i) leaf extract (EE Leaves): dominated by flavonoid glycosides including kaempferol‐*O*‐glucoside (5, 13.1%), 6‐hydroxyluteolin‐*O*‐glucoside (6, 1.49%), and apigenin‐*O*‐glucuronide (4, 2.98%). The leaf extract also contained high concentrations of Mansoin A–type compounds (41, 41.84%; 49, 48.42%), characterized as dihydrochalcone‐flavanone heterotrimers (*m/z* 1259.4231 [M + H]^+^, Figure 1). Additionally, dihydrochalcone‐flavone conjugates (24–27) and dihydrochalcone‐flavonol conjugates (30–31) were predominantly detected in leaves, suggesting leaf tissue as the primary site for complex oligomeric flavonoid biosynthesis; (ii) stem extract (EE Stems): characterized by the presence of phenylethanoid glycosides, particularly verbascoside (17, 13.9%) and isoverbascoside (18, 4.07%), along with cinnamoyl substituted mangiferin derivatives (14–16). The stem extract also contained dihydrochalcone‐tyramine conjugates (9–11) with moderate abundances (1.36%–11.77%), indicating distinct biosynthetic pathways in stem tissues compared to leaves and fruits; (iii) fruit extract (EE Fruits) exhibited the highest diversity of simple phenolic precursors, including 2′,4′,4‐trihydroxydihydrochalcone (1, 3.44%) and eriodictyol (2, 2.8%). The fruits accumulated significant amounts of dihydrochalcone‐tyramine conjugates (9, 20.48%; 11, 32.54%) and Mansoin *C*‐type compounds (19, 16.19%; 22, 16.30%), suggesting preferential accumulation of specific conjugation products in reproductive tissues.

### 3.2 Characterization of Flavonoid Conjugates Containing Dihydrochalcone and Tyramine Moieties

Eleven compounds incorporating tyramine (4‐hydroxyphenethylamine) residues conjugated with dihydrochalcone units were putatively identified in *M. hirsuta* extracts (Table 1). These metabolites represent a distinctive class of nitrogen‐containing phenolic conjugates characterized by a characteristic neutral loss of 137 Da (C_8_H_9_NO), corresponding to the cleavage of the tyramine residue [21]. The presence of a single nitrogen atom in these compounds is confirmed by the nitrogen rule [22, 23]. All compounds exhibit odd nominal masses (*m/z* 380.1861 [M + H]^+^, 526.2439 [M + H]^+^, 542.2393 [M + H]^+^, 946.3861 [M + H]^+^, 1004.3550 [M + H]^+^), consistent with the presence of an odd number of nitrogen atoms (*n* = 1) in their molecular formulas. This structural feature distinguishes them from the nitrogen‐free flavonoid conjugates discussed in preceding sections.

Compound **3** (at *m/z* 380.1861 [M + H]^+^, C_23_H_25_NO_4_, RT 12.5 min) represents the simplest member of this series, detected exclusively in fruit extracts (Figures 2 and S2). The MS^2^ fragmentation revealed a base peak at *m/z* 243.1018 [M + H]^+^, corresponding to the protonated dihydrochalcone aglycone (C_15_H_11_O_4_^+^), with a neutral loss of 137 Da, consistent with the elimination of the tyramine moiety (C_8_H_11_NO) [21]. Additional fragment ions at *m/z* 138.0915 [M + H]^+^ (13.5%) and *m/z* 137.0597 [M + H]^+^ (14.1%) correspond to the protonated tyramine cation and the fragment ion of the resorcinol moiety of the base peak, respectively, confirming the structural assignment. The UV absorption profile (*λ*_max_ 219 nm) is characteristic of dihydrochalcone chromophores.

**FIGURE 2:**
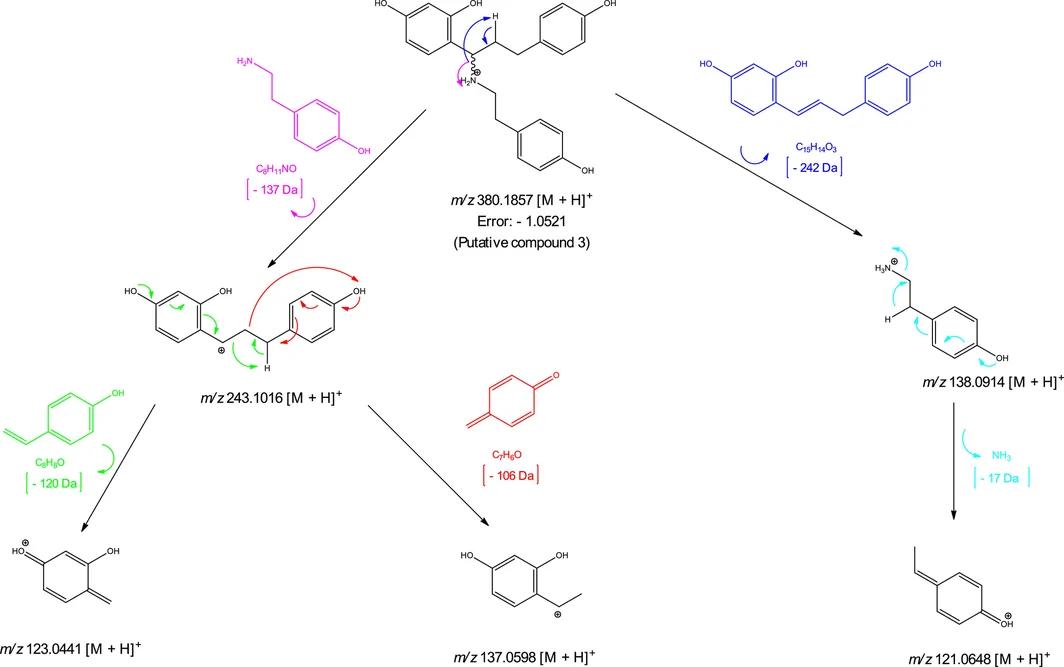
Proposed fragmentation pathways of ion *m/z* 380. 1857 [M + H]^+^, putatively identified as compound 3, a conjugate of dihydrochalcone and tyramine moieties, detected in the *M. hirsuta* extracts.

The compounds with putative molecular formulas C_29_H_35_NO_8_ (*m/z* 526.2438–526.2439 [M + H]^+^, compounds **7–8**) (Figure S3) and C_29_H_35_NO_9_ (*m/z* 542.2393–542.2395 [M + H]^+^, compounds **9–12**) (Figure S4) were detected, representing glycosylated derivatives of the basic dihydrochalcone‐tyramine scaffold. The MS^2^ fragmentation of the compounds **7–8** revealed base peaks at *m/z* 243.1016–243.1017 [M + H]^+^, with secondary fragment ions at *m/z* 149.0568, 137.0596, 133.0647, and 123.0439 [M + H]^+^, consistent with cleavages within the tyramine and sugar moieties. Already, the compounds **9–12** (RT 11.5–12.2 min) exhibit the molecular formula C_29_H_35_NO_9_, corresponding to an additional oxygen atom (16 Da) compared to compounds **7–8**, suggesting hydroxylation in the putative compound. The MS^2^ fragmentation of compounds **9–12** consistently showed neutral losses of 137 Da (tyramine) and shared fragment ions at *m/z* 405.1548 (aglycone after tyramine loss) and *m/z* 243.1020 [M + H]^+^ (dihydrochalcone base peak), supporting the putative proposed structure (C_29_H_35_NO_9_) with substitution in the sugar residues. These four isomers displayed distinct tissue distribution patterns: compounds 9 and 11 were predominantly detected in stems and fruits, whereas compound 10 showed lower abundance in both tissues, and compound 12 was restricted to fruits. This differential distribution suggests positional isomerism of the glycosyl substituent or variations in the hydroxylation pattern.

Two higher molecular mass compounds (C_50_H_60_NO_17_) with ions at *m/z* 946.3863 [M + H]^+^ (compound **33**) and *m/z* 946.3861 [M + H]^+^ (compound **34**) were detected at retention times 13.9 and 14.4 min, exclusively in the fruit extracts. The molecular formulas of the isomers indicate putative trimeric structures comprising two dihydrochalcone units, one tyramine residue, and multiple glycosyl substituents. The MS^2^ fragmentation revealed sequential losses of tyramine (137 Da) and two hexose units (2 × 162 Da), affording fragment ions at *m/z* 809.3015/809.2941 [M + H − 137]^+^, *m/z* 647.2497/647.2505 [M + H − 137 − 162]^+^, and *m/z* 485.1956 [M + H − 137 − 162 − 162]^+^. Additional ions at *m/z* 375.1594 [M + H]^+^, 269.1176 [M + H]^+^, and 133.0646/133.0645 [M + H]^+^ reflect cleavages within the dihydrochalcone dimeric core and the tyramine residue. The base peak at *m/z* 243.1017–243.1018 [M + H]^+^ confirms the presence of the dihydrochalcone structural unit as the fundamental building block of these oligomeric conjugates (Figure S5).

Moreover, two isomers with ions at *m/z* 1004.3550 [M + H]^+^ and 1004.3557 [M + H]^+^ (C_51_H_57_NO_20_) were detected in fruit extracts (RT 14.7 and 14.9 min). These compounds represent putative complex hybrid structures, containing dihydrochalcone, flavanone, and tyramine moieties. The MS^2^ fragmentation revealed sequential losses of tyramine (137 Da) sugar and resorcinol residues, respectively, affording fragment ions at *m/z* 867.2707 [M + H − 137]^+^, *m/z* 705.2174/705.2173 [M + H − 137 − 162]^+^, and *m/z* 595.1809 [M + H − 137 − 162 − 110]^+^ (base peak). Additional fragment ions at *m/z* 433.1284 [M + H]^+^, 327.0863 [M + H]^+^, and 297.0760/297.0759 [M + H]^+^ support the proposed structures involving a flavanone unit (characteristic fragment ions at *m/z* 327 and 297 [M + H]^+^) conjugated with dihydrochalcone and tyramine residues (Figure S6). The presence of the flavanone moiety distinguishes these compounds from the simpler dihydrochalcone‐tyramine conjugates and aligns them with the biosynthetic pathway leading to the Mansoin‐type flavonoid conjugates.

The structural diversity of tyramine‐containing conjugates in *M. hirsuta* suggests a biosynthetic pathway involving the conjugation of tyramine (derived from tyrosine via tyrosine decarboxylase) with dihydrochalcone units activated as CoA esters, catalyzed by tyramine *N*‐hydroxycinnamoyl transferase (THT) or related enzymes [24]. The presence of glycosylated and oligomeric forms indicates subsequent modifications by glycosyltransferases and potential oxidative coupling reactions. The tissue‐specific accumulation of these compounds in fruits (compounds **3**, **7–8**, **12**, **33–34**, **36–37**) and stems (compounds **9–11**) suggests differential expression of the biosynthetic machinery across plant organs, with fruits serving as the primary site for the accumulation of complex tyramine‐containing conjugates [24].

### 3.3 Characterization of Flavonoid Conjugates Containing Dihydrochalcone and Flavanone Moieties

Among the 57 polyphenols putatively identified by LC‐DAD‐HRMS (Table 1), 19 compounds were characterized as flavonoid conjugates incorporating a central flavanone residue. This structural class includes the previously described mansoins A‐F [8, 9], alongside 13 novel or structurally related analogues. The diagnostic fragmentation pattern of these conjugates is characterized by the presence of a base peak at *m/z* 243.1018 [M + H]^+^, corresponding putatively to the protonated dihydrochalcone aglycone (C_15_H_11_O_4_^+^), which serves as a key reporter ion for this structural class. The neutral losses of hexose residues (162 Da) and the characteristic UV absorption profiles (*λ*_max_ ~ 196–220 nm and 275–285 nm) further support the structural assignments.

Indeed, the MS^2^ fragmentation of dihydrochalcone‐flavanone conjugates follows predictable patterns that enable structural characterization. The cleavage of the interflavonoid C–C bond between the dihydrochalcone β'‐carbon and the flavanone A‐ring generates the ion diagnostic at *m/z* 243.1018 [M + H]^+^ (protonated 2′,4′,4‐trihydroxydihydrochalcone), whereas the complementary fragment retains the flavanone moiety plus glycosyl substituents. For glycosylated conjugates, sequential neutral losses of 162 Da (hexose) are observed, with the glycosidic bond cleavage occurring preferentially at the flavanone 3‐OH or 7‐OH positions, based on the characteristic fragment ions observed [25]. Furthermore, the UV absorption profiles of these conjugates (Band II: 196–220 nm; Band I: 275–285 nm) are consistent with the presence of dihydrochalcone chromophores, which lack the extended conjugation observed in chalcones (*λ*_max_ 340–390 nm) [26].

The dihydrochalcone‐flavanone conjugates detected in *M. hirsuta* span a wide molecular weight range, from monoglycosylated dimers (*m/z* 677.2237–693.2180 [M + H]^+^) to complex oligomeric structures (*m/z* 1097.3680 − 1421.4750 [M + H]^+^). Representative precursor ions were observed at *m/z* 677.2237 [M + H]^+^ (compound **19**, C_36_H_36_O_13_) (Figure S7), *m/z* 693.2180 [M + H]^+^ (compound **21**, C_36_H_36_O_14_) (Figure S8), and *m/z* 839.2773 [M + H]^+^ (compound **22**, C_42_H_46_O_18_) (Figure S9), each representing distinct glycosylation patterns and aglycone compositions. As for the compounds **28** and **29**, isomers of molecular ion at *m/z* 855.2727 [M + H]^+^ (C_42_H_46_O_19_), were putative identified in a manner similar to the compound **22**, with the presence of a catechol system in *C*‐ring (Figure S10). Notably, compound **19** (RT 19.2 min) exhibited a characteristic neutral loss of 162 Da, generating a fragment ion at *m/z* 515.1704 [M + H]^+^, consistent with the elimination of a hexosyl residue. The base peak at *m/z* 405.1336 [M + H]^+^, accompanied by secondary fragment ions at *m/z* 299.0918, 285.0762, 243.1020, and 179.0341 [M + H]^+^ supports the assignment of a glycosylated heterodimer comprising one dihydrochalcone and one flavanone unit (Figure S7). The fragmentation pathway, involving sequential cleavages of the glycosidic bond and Retro–Diels–Alder (RDA) rearrangements of the flavanone *C*‐ring, is consistent with previously reported fragmentation mechanisms for biflavonoid structures [25].

The mass spectrum revealed the presence of higher molecular mass conjugates, including compounds **32** (*m/z* 935.3133 [M + H]^+^, C_51_H_50_O_17_) (Figure S11), and the isomers **39**–**42** (*m/z* 1097.3680 [M + H]^+^, C_57_H_60_O_22_) (Figure S12), putatively identified as isomers of Mansoin B type, and compounds **47**–**51** (*m/z* 1259.4157 [M + H]^+^, C_63_H_70_O_27_), corresponding to Mansoin A and its isomers. Compounds at *m/z* 935.3133 and *m/z* 1097.3680 [M + H]^+^ differ by 162 Da, indicating the difference of a hexose residue. These compounds share a common fragmentation fingerprint, including the diagnostic base peak at *m/z* 243.1018 [M + H]^+^ and fragment ions at *m/z* 693.2170, 553.1852, 421.1279, and 405.1541 [M + H]^+^, indicative of the conserved dihydrochalcone‐flavanone core structure. Mansoin A (compound **49**, RT 18.8 min) was the most abundant isomer in the leaf extract, displaying the characteristic fragment ions at *m/z* 1097.3680, 987.3295, 855.2707, and 693.2173 [M + H]^+^ (Figure 1), consistent with sequential losses of sugar residues and the dihydrochalcone unit.

Two isomers at *m/z* 1339.4651 [M + H]^+^ (compounds **54** and **55**, C_72_H_74_O_25_) represent structural variants of Mansoin A containing an additional dihydrochalcone residue, as evidenced by the enhanced relative intensity of the *m/z* 243.1018 [M + H]^+^ fragment and the presence of ions at *m/z* 647.2463 and 485.1943 [M + H]^+^, corresponding to the sequential loss of two dihydrochalcone units (Figure S13). Similarly, compounds **56** and **57** (*m/z* 1421.4750 [M + H]^+^, C_69_H_80_O_32_) differ from Mansoin A by the incorporation of an additional hexose residue, as confirmed by the neutral loss of 162 Da from the precursor ion and the presence of fragment ions at *m/z* 855.2721 and 693.2183 [M + H]^+^ (Figure S14).

Finally, the dihydrochalcone‐flavanone conjugates exhibited distinct tissue distribution patterns. Compounds **19** and **22** were detected in the fruit extract, whereas Mansoin A and related isomers (**47**–**51**) were exclusively or predominantly found in leaf extracts. This chemotaxonomic differentiation suggests tissue‐specific biosynthesis pathways for these specialized metabolites in *M. hirsuta* .

### 3.4 Characterization of Flavonoid Conjugates Containing Dihydrochalcone and Flavan‐3‐ol Moieties

Among the 57 polyphenols identified in *M. hirsuta* extracts, two novel flavonoid conjugates incorporating dihydrochalcone and flavan‐3‐ol structural units were putatively detected (compounds **20** and **23**, Table 1). These heterodimeric structures represent a distinct subclass of biflavonoid conjugates characterized by the presence of a saturated *C*‐ring (flavan‐3‐ol moiety) rather than the unsaturated *C*‐ring observed in flavanone‐containing analogues (compounds **19**, **21–22**, **28–29**, **32**, **39–42**, **48–52**, **55–56**, **57–58**).

Compound **20** exhibited a protonated molecular ion at *m/z* 679.2387 [M + H]^+^ (C_36_H_38_O_13_) with a retention time of 18.3 min (Figures 3 and S15). The ion at *m/z* 517.1849 [M + H − 162]^+^is consistent with the presence of one hexoside residue. The base peak at *m/z* 407.1486 [M + H]^+^ corresponds to the protonated aglycone resulting from cleavage of the glycosidic bond and subsequent rearrangement. The diagnostic fragment ion at *m/z* 243.1007 [M + H]^+^ (8.0%) confirms the presence of the dihydrochalcone unit (C_15_H_11_O_4_^+^), whereas fragment ions at *m/z* 301.1070 (60.0%), 287.0919 (7.8%), 195.0650 (18.1%), and 181.0495 [M + H]^+^ (6.7%) originate from the flavan‐3‐ol moiety through characteristic RDA and Heterocyclic Ring Fission (HRF) cleavages [27]. The fragment ion at *m/z* 301.1070 [M + H]^+^ corresponds to the protonated flavan‐3‐ol aglycone (C_15_H_13_O_6_^+^), likely representing (*epi*)catechin or another stereoisomer, formed through Quinone Methide (QM) cleavage of the interflavonoid bond [27]. The ion at *m/z* 287.0919 [M + H]^+^ suggests dehydration of the flavan‐3‐ol unit, whereas fragments at *m/z* 195.0650 and 181.0495 [M + H]^+^ correspond to RDA cleavage products involving the *B*‐ring and *C*‐ring of the flavan‐3‐ol moiety [28].

**FIGURE 3:**
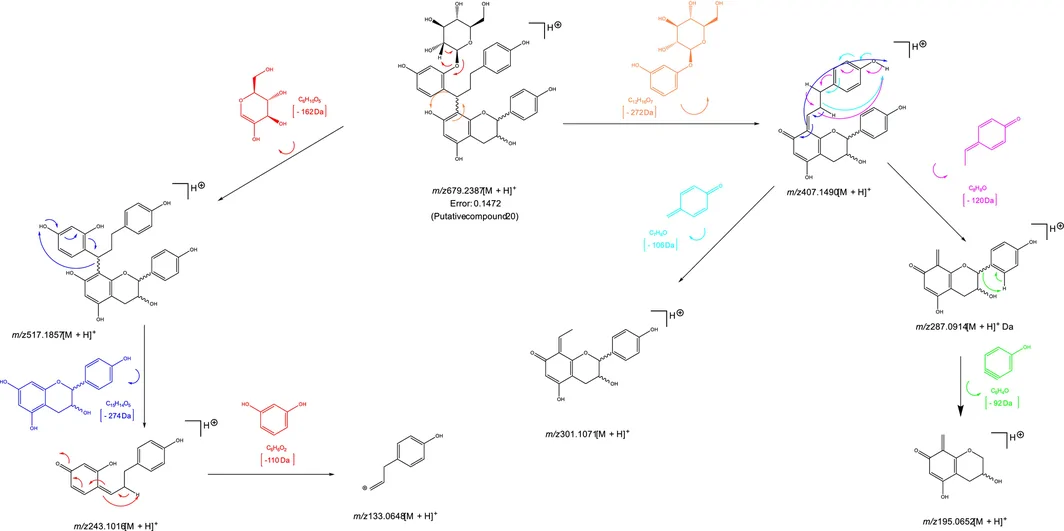
Proposed fragmentation pathways of ion *m/z* 679.2387 [M + H]^+^, putatively as compound 20, a conjugate of dihydrochalcone and flavan‐3‐ol moieties, detected in the *M. hirsuta* extracts.

Compound **23** (at *m/z* 841.2929 [M + H]^+^, C_42_H_48_O_18_, RT 18.5 min) represents a diglycosylated analogue of compound **20**, as evidenced by the molecular mass difference of 162 Da (C_6_H_10_O_5_, hexose) (Figure S16). This compound was detected exclusively in leaf extracts, co‐eluting with compound **20** at a similar retention time, suggesting possible co‐occurrence as biosynthetic relatives. Indeed, the MS^2^ fragmentation of compound **23** revealed sequential neutral losses of two hexose residues (2 × 162 Da), generating fragment ions at *m/z* 679.2379 [M + H − 162]^+^ and *m/z* 517.1851 [M + H − 162 − 162]^+^. The base peak at *m/z* 407.1488 [M + H]^+^ corresponds to the protonated dihydrochalcone‐flavan‐3‐ol aglycone, identical to that observed for compound **20**. The presence of the diagnostic dihydrochalcone fragment at *m/z* 243.1017 [M + H]^+^ (21.5%), along with flavan‐3‐ol characteristic ions at *m/z* 301.1070 (48.1%) and 195.0654 [M + H]^+^ (9.8%), confirms the structural homology between both compounds, differing only in the degree of glycosylation. The UV absorption profile (*λ*_max_ 197, 220, 284 nm) is consistent with the presence of dihydrochalcone chromophores lacking the extended conjugation of chalcones [26].

The co‐occurrence of compounds **20** and **23** in leaf extracts suggests a common biosynthetic origin, where compound **23** likely represents the glycosylated precursor or derivative of compound **20**. The structural relationship between these flavan‐3‐ol conjugates and the flavanone‐containing analogues (e.g., compounds **19** and **21**) indicates a versatile biosynthetic pathway in *M. hirsuta* capable of generating diverse *C–C*‐linked biflavonoid structures with different oxidation states of the *C*‐ring. The absence of these compounds in stem extracts highlights the tissue‐specific accumulation of dihydrochalcone‐flavan‐3‐ol conjugates in *M. hirsuta* , contrasting with the broader distribution of flavanone‐containing analogues. This chemotaxonomic pattern may reflect distinct enzymatic activities (e.g., flavanone 3‐hydroxylase vs. dihydroflavonol 4‐reductase) governing the biosynthesis of flavan‐3‐ol vs. flavanone units in different plant organs [28].

### 3.5 Characterization of Flavonoid Conjugates Containing Dihydrochalcone and Flavone Moieties

Ten compounds incorporating a central flavone moiety (C_2_ = C_3_ unsaturated in the *C*‐ring) were putatively identified in *M. hirsuta* extracts (Table 1). This structural class exhibits distinct UV absorption profiles compared to flavanone‐containing analogues, with characteristic Band I absorption at 273–280 nm due to the extended conjugation of the *C*‐ring [29]. The flavone‐based conjugates span molecular mass ranges from diglycosylated dimers (*m/z* 853.2552–853.2564 [M + H]^+^, C_42_H_44_O_19_) to complex oligomeric structures (*m/z* 1257, C_63_H_68_O_27_). A proposed fragmentation pathway of ion *m/z* 853.2562 [M + H]^+^, for isomer compounds 24–27, is represented in Figure 4 (Figure S17). The MS^2^ fragmentation revealed sequential neutral losses of 162 Da (at *m/z* 691.2017 [M + H − 162]^+^ and at *m/z* 529.1489 [M + H − 162 − 162]^+^), consistent with the elimination of two glycosyl residues. Already the fragment ions at *m/z* 419.1124 [M + H]^+^ (C_21_H_19_O_9_^+^) and *m/z* 313.0707 [M + H]^+^ (C_16_H_13_O_7_^+^) are diagnostic of the flavone moiety. These fragments result from RDA cleavage of the *C*‐ring, characteristic of flavone aglycones [29]. The ion at *m/z* 419.1124 [M + H]^+^ represents the QM fragmentation of the dihydrochalcone, whereas fragment ion *m/z* 313.0707 [M + H]^+^ corresponds to subsequent losses from the *B*‐ring [30]. The presence of these fragments, distinct from the flavanone fragmentation pattern, confirms the unsaturated *C*‐ring structure of the flavone unit [29].

**FIGURE 4:**
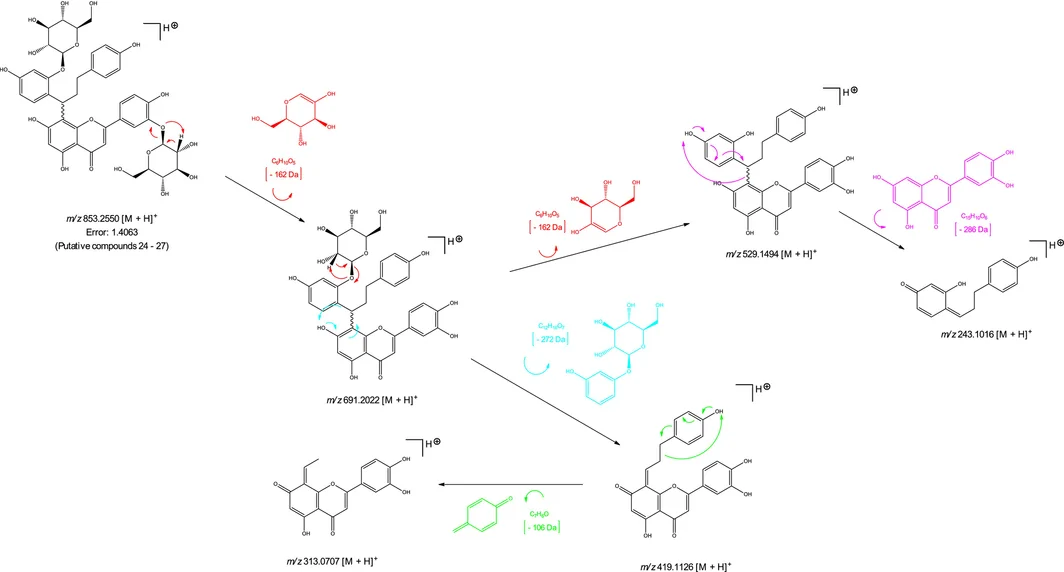
Proposed fragmentation pathways of ion *m/z* 853.2562 [M + H]^+^, putatively identified as isomers of conjugate dihydrochalcone and flavone moieties, detected in the *M. hirsuta* extracts. Putative isomers of compounds 24, 25, 26, and 27.

Compound **35** (at *m/z* 999.2938 [M + H]^+^, C_51_H_50_O_21_; RT 17.3 min) represents a putative structurally distinct flavone conjugate incorporating a caffeic acid residue (Figure S18). A neutral loss of 308 Da was observed, corresponding to the elimination of a caffeic acid (146 Da) glucose (162 Da) residues. The fragment at *m/z* 529.1494 [M + H − 146 − 162 − 162]^+^ corresponds to the dihydrochalcone‐flavone dimeric core, whereas the base peak at *m/z* 147.0438 [M + H]^+^ is attributed to the protonated caffeic acid residue by ion acylium fragmentation (C_9_H_7_O_4_^+^), confirming the presence of this hydroxycinnamic acid substituent.

Furthermore, a peak at *m/z* 1067.3681 [M + H]^+^ (C_60_H_58_O_18_) was detected at retention time 20.7 min exclusively in leaf extracts (Figure S19). The MS^2^ fragmentation revealed a prominent ion at *m/z* 663.2193 [M + H]^+^ (54.2%), putatively attributed to the cleavage between *C*‐8 of the flavone residue and *C*‐β' of a dihydrochalcone unit. The base peak at *m/z* 243.1024 [M + H]^+^ confirms the presence of the dihydrochalcone structural unit, whereas secondary fragment ions at *m/z* 557.1815, 553.1851, and 531.1666 [M + H]^+^ support a proposed structure comprising three dihydrochalcone residues and one flavone unit with an additional hexose substituent.

Finally, four peaks at *m/z* 1257.4067–1257.4072 [M + H]^+^ were detected in the EIC between retention times 15.9–17.4 min (compounds **43–46**). These isomers share the molecular formula C_63_H_68_O_27_ and exhibit similar fragmentation patterns involving sequential neutral losses of 162 Da (at *m/z* 1095.3413 [M + H − 162]^+^ and at *m/z* 933.2954 [M + H − 162 − 162]^+^) (Figure S20). The base peak at *m/z* 243.1014–243.1018 [M + H]^+^ and secondary fragment ions at *m/z* 771.2426 [M + H − 162 − 162 − 162]^+^, and *m/z* 419.1125 [M + H − 162 − 162 − 162 − 242 − 110]^+^ confirm the conserved dihydrochalcone‐flavone core structure with variable glycosylation patterns.

The structural distinction between flavone and flavanone conjugates is exemplified by comparison with compounds **39–42** (Mansoin B type, at *m/z* 1097.3673–1097.3680 [M + H]^+^). Although these compounds share similar molecular mass with the flavone series, their UV absorption profiles (*λ*_max_ ~ 284 nm without pronounced Band I) and fragmentation patterns (absence of characteristic flavone RDA fragments at *m/z* 313.0704 [M + H]^+^) enable clear differentiation [29]. The unsaturated *C*‐ring of flavones (compounds **24–27**, **35**, **38**, **43–46**) results in distinct MS^2^ behavior compared to the saturated *C*‐ring of flavanones, with flavones typically exhibiting enhanced ^1,3^A^+^ and ^1,3^B^+^ fragment ions due to the conjugated system [30].

### 3.6 Characterization of Flavonoid Conjugates Containing Dihydrochalcone and Flavonol Moieties

Four compounds incorporating a central flavonol (3‐hydroxyflavone) moiety were putatively identified in *M. hirsuta* leaf extracts (Table 1). Flavonols are structurally distinguished from flavones by the presence of a hydroxyl group at the C3 position in addition to the C_2_ = C_3_ double bond in the *C*‐ring [31]. This structural feature confers distinct UV absorption profiles, with flavonols typically exhibiting Band I at 300–380 nm (due to the *B*‐ring cinnamoyl system) and Band II at 240–280 nm (*A*‐ring benzoyl system), with the exact position of Band I depending on the hydroxylation pattern of the *B*‐ring [32].

Two isomers with ions at *m/z* 869.2508 and 869.2512 [M + H]^+^ (compounds **30** and **31**, respectively; C_42_H_44_O_20_) were detected at retention times 15.5 and 15.9 min (Figures 5 and S21). These compounds share the molecular formula with the flavone analogues (compounds **24–27**), but with an additional oxygen atom (16 Da), consistent with the presence of a 3‐hydroxyl group on the *C*‐ring (flavonol vs. flavone) [32].

**FIGURE 5:**
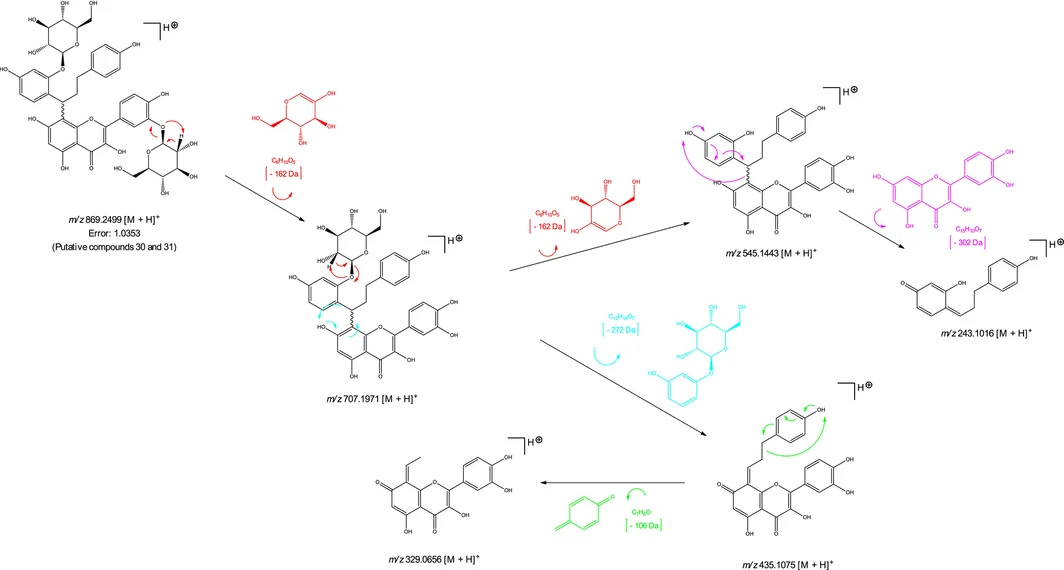
Proposed fragmentation pathways of ion *m/z* 869.2508 [M + H]^+^, putatively identified as isomers of conjugate dihydrochalcone and flavonol moieties, detected in the *M. hirsuta* extracts. Putative isomers of compounds 30 and 31.

The MS^2^ fragmentation revealed sequential neutral losses of two hexose residues (2 × 162 Da), generating fragment ions at *m/z* 707.1962 [M + H − 162]^+^ and *m/z* 545.1436 [M + H − 162 − 162]^+^. The base peak at *m/z* 545.1436–545.1439 corresponds to the protonated dihydrochalcone‐flavonol aglycone. Characteristic fragment ions at *m/z* 435.1070–435.1072 [M + H]^+^ (C_21_H_19_O_10_^+^) and *m/z* 329.0655–329.0657 [M + H]^+^ (C_16_H_13_O_8_^+^) are diagnostic of the flavonol moiety. These fragments differ from those observed in flavone analogues by the presence of the additional hydroxyl group, as confirmed by accurate mass measurements. The diagnostic dihydrochalcone fragment at *m/z* 243.1016–243.1018 [M + H]^+^ confirms the presence of the dihydrochalcone structural unit. The UV absorption profiles (*λ*_max_ 199, 220, 276 nm for compound **30**; 197, 220, 273 nm for compound **31**) are consistent with flavonol chromophores, exhibiting a characteristic shoulder at ~270–280 nm and a less pronounced Band I compared to fully conjugated flavones, due to the electron‐withdrawing effect of the 3‐OH group [32].

Two additional isomers with ions at *m/z* 1273.4019 and 1273.4028 [M + H]^+^ (compounds **52** and **53**; C_63_H_68_O_28_) were detected at retention times 15.6 and 15.7 min (Figure S22). These compounds represent higher molecular mass analogues of compounds **30–31**, incorporating an additional hexose residue (162 Da) and an extra dihydrochalcone or flavonol unit. The MS^2^ fragmentation revealed sequential neutral losses of three hexose residues (3 × 162 Da), generating fragments at *m/z* 1111.3469 [M + H − 162]^+^, *m/z* 949.2995 [M + H − 162 − 162]^+^, and *m/z* 787.2385 [M + H − 162 − 162 − 162]^+^. The cleavage between C‐β' of the dihydrochalcone residue and C‐8 of the flavonol moiety leads to the formation of characteristic ions at *m/z* 303.0496 [M + H − 162 − 162 − 162 − 242 − 242]^+^ (protonated flavonol aglycone, C₁₅H₁₁O₆^+^) [30]. The base peak at *m/z* 243.1015–243.1018 [M + H]^+^ confirms the dihydrochalcone structural residue, whereas fragments at *m/z* 435.1069–435.1072 [M + H]^+^ (73.4%–86.9% relative abundance) and *m/z* 329.0651–329.0653 [M + H]^+^ (74.4%–79.8%) are diagnostic of the flavonol moiety, consistent with the fragmentation patterns observed for compounds **30–31**. The structural distinction between flavonol, flavone, and flavanone conjugates is exemplified by comparison with compounds **24–27** (flavones, C_42_H_44_O_19_, *m/z* 853.2562–853.2564 [M + H]^+^) (Figure S17) and compounds **28–29** (flavanones, C_42_H_46_O_19_, *m/z* 855.2720–855.2717 [M + H]^+^) (Figure S10). The accurate mass difference of 16 Da between flavonol (C_42_H_44_O_20_) and flavone (C_42_H_44_O_19_) analogues corresponds to one oxygen atom, whereas the 2‐Da difference between flavone and flavanone corresponds to two hydrogen atoms (C_2_ = C_3_ vs. C_2_–C_3_) [32].

### 3.7 Identification of Additional Polyphenolic Compounds

The co‐occurrence of dihydrochalcone‐flavonol (compounds **30–31**, **52–53**), dihydrochalcone‐flavone (compounds **24–27**, **35**, **38**, **43–46**), and dihydrochalcone‐flavanone (compounds **19**, **21–22**, **28–29**, **32**, **39–42**, **47–51**, **54–55**, **56–57**) conjugates in *M. hirsuta* demonstrates the versatility of the flavonoid biosynthetic pathway in this species. Beyond the complex flavonoid conjugates characterized in the preceding sections, the LC‐DAD‐HRMS analysis of the leaf, stem, and fruit EEs from *M. hirsuta* revealed the presence of 12 additional polyphenolic compounds, including simple flavonoids, acylated xanthone *C*‐glycosides, and phenylethanoid glycosides (Table 1). These compounds were identified based on their accurate mass values, characteristic UV absorption profiles, tandem MS fragmentation patterns, and retention times, in comparison with authentic reference standards and literature data from previous phytochemical investigations of Bignoniaceae species [33].

The crude extracts contained several monomeric flavonoids representing key biosynthetic precursors of the more complex conjugates. Compound **1** (2′,4′,4‐trihydroxydihydrochalcone; at *m/z* 259.0967 [M + H]^+^, C_15_H_14_O_4_) (Figure S23) was detected exclusively in fruit extracts and represents the fundamental dihydrochalcone unit found in all biflavonoid conjugates characterized in this study. Its UV spectrum (*λ*_max_ 219, 276 nm) and MS^2^ fragmentation pattern, showing characteristic ions at *m/z* 197.1179 and 153.0544 [M + H]^+^, are consistent with the structure of phloretin derivatives [34]. Already, the compound **2** (eriodictyol; at *m/z* 289.0708 [M + H]^+^, C_15_H_12_O_6_) was identified as a flavanone aglycone based on its UV absorption (*λ*_max_ 219, 276 nm) and diagnostic MS^2^ fragments at *m/z* 163.0393 and 153.0183 [M + H]^+^, resulting from RDA cleavage of the C‐ring [30] (Figure S24). This compound was detected in fruit extracts and represents the basic structural unit of the flavanone‐containing conjugates.

Three flavonoid *O*‐glycosides were identified in leaf extracts: compound **4** (apigenin‐*O*‐glucuronide; at *m/z* 447.0919 [M + H]^+^) (Figure S25), compound **5** (kaempferol‐*O*‐glucoside; at *m/z* 449.1079 [M + H]^+^) (Figure S26), and compound **6** (6‐hydroxyluteolin‐*O*‐glucoside; at *m/z* 465.1023 [M + H]^+^) (Figure S27). These compounds exhibited characteristic UV spectra of flavones (*λ*_max_ ~ 197–220, 274–352 nm) and neutral losses of 176 Da (glucuronic acid) or 162 Da (glucose), consistent with *O*‐glycosidic linkages at the 7‐OH or 3‐OH positions [29].

Furthermore, four structurally related xanthone derivatives were detected exclusively in stem extracts, representing a distinct class of specialized metabolites in *M. hirsuta* . These compounds are *C*‐glycosides of mangiferin (1,3,6,7‐tetrahydroxyxanthone‐2‐*C*‐glucoside) with different hydroxycinnamic acid acylations at the 2′‐*O*‐position of the *C*‐bound glucose moiety [35]. Thus, the compound **13** (muraxanthone, 2′‐*O*‐*p*‐hydroxybenzoylmangiferin; at *m/z* 543.1113 [M + H]^+^, C_26_H_22_O_13_) (Figure S28), exhibited a base peak at *m/z* 285.0385 [M + H]^+^ (100%) corresponding to the protonated mangiferin aglycone, with the neutral loss of 258 Da (*p*‐hydroxybenzoic acid + glucose rearrangement) [36]. Compounds **14–16** showed progressive increases in molecular mass of 16 Da, corresponding to methoxy (cinnamoyl), hydroxymethoxy (coumaroyl), and dihydroxymethoxy (caffeoyl) substitutions, respectively [35]. Compound **14** (2′‐*O*‐trans‐cinnamoylmangiferin; at *m/z* 553.1318 [M + H]^+^) displayed characteristic fragments at *m/z* 433.0901 [M + H]^+^ (loss of cinnamoyl, 120 Da) and *m/z* 131.0483 [M + H]^+^ (protonated cinnamic acid) (Figure S29). Compound **15** (2′‐*O*‐trans‐coumaroylmangiferin; at *m/z* 569.1271 [M + H]^+^) showed a base peak at *m/z* 147.0434 [M + H]^+^ (protonated coumaric acid) (Figure S30), whereas compound **16** (2′‐*O*‐trans‐caffeoylmangiferin; at *m/z* 585.1215 [M + H]^+^) (Figure S31), exhibited the characteristic caffeic acid fragment ion at *m/z* 163.0385 [M + H]^+^ (100%) [35]. The UV spectra of these compounds (*λ*_max_ ~ 195–225, 280–325, 353 nm) are consistent with the extended conjugation introduced by the hydroxycinnamoyl substituents [35].

Two isomeric phenylethanoid glycosides were identified in stem extracts: compound **17** (verbascoside, acteoside; at *m/z* 625.2103 [M + H]^+^) (Figure S32) and compound **18** (isoverbascoside; at *m/z* 625.2101 [M + H]^+^) (Figure S33). These compounds share the molecular formula C_29_H_36_O_15_ and exhibit characteristic UV absorption at *λ*_max_ 199, 326 nm (compound **17**) and 220, 323 nm (compound **18**), consistent with the caffeoyl and hydroxytyrosol chromophores [34].

The MS^2^ fragmentation of verbascoside and isoverbascoside revealed diagnostic ions at *m/z* 479.1527/479.1526 [M + H − 146 (rhamnose)]^+^, *m/z* 325.0907/325.0906 [M + H − 138 (hydroxytyrosol) − 162 (glucose)]^+^, and the base peak at *m/z* 163.0383/163.0384 [M + H]^+^ (protonated caffeic acid) [40]. The differentiation between these positional isomers is based on their distinct retention times (14.2 min for verbascoside vs. 14.8 min for isoverbascoside) and subtle differences in fragment ion abundances, consistent with literature reports [36].

The distribution of these polyphenolic compounds across plant tissues reveals distinct biosynthetic patterns. Simple flavonoids (compounds **1–2**) accumulate predominantly in fruits, whereas flavonoid glycosides (compounds **4–6**) and phenylethanoid glycosides (compounds **17–18**) are concentrated in leaves and stems, respectively. The acylated xanthone *C*‐glycosides (compounds **13–16**) represent stem‐specific metabolites, suggesting tissue‐specific expression of the acyltransferases responsible for the decoration of the mangiferin core structure [35]. These findings align with previous phytochemical investigations of Bignoniaceae species from the Brazilian Cerrado, which have documented the occurrence of mangiferin derivatives and phenylethanoid glycosides as chemotaxonomic markers of the family [33]. The co‐occurrence of these compounds with the complex flavonoid conjugates characterized in this study underscores the metabolic diversity of *M. hirsuta* and its potential as a source of bioactive polyphenols.

### 3.8 Antiviral Assays

All extracts showed low cytotoxicity in LLC MK2 and Vero cells (CC_50_ > 400–500 μg mL^−1^), providing favorable therapeutic windows (Table 2). The stem extract demonstrated potent and selective anti‐ZIKV activity (EC_50_ 15.7 ± 1.9 μg mL^−1^, SI > 25.5), markedly exceeding the promising threshold (SI ≥ 10). Moderate activity was observed against CHIKV (EC_50_ 154.5 ± 3.8 μg mL^−1^, SI > 2.6) and MAYV (EC_50_ 101.1 ± 3.2 μg mL^−1^, SI > 4.0). The leaf extract exhibited broad‐spectrum activity, with notable SI values against ZIKV (10.3), CHIKV (4.2), and MAYV (4.1), with its EC_50_ against ZIKV (34.6 ± 1.7 μg mL^−1^) compared favorably to ribavirin (EC_50_ 94.47 μg mL^−1^, SI 3.9). The fruit extract showed the highest potency against DENV (EC_50_ 25.4 ± 1.2 μg mL^−1^, SI > 19.7) and significant activity against ZIKV (SI > 14.1) and MAYV (SI > 9.5).

**TABLE 2:** Cytotoxic activity (CC_50_) and evaluation of antiviral activity (EC_50_) against DENV 2, ZIKV, CHIKV, and MAYV in LLCMK‐2 and Vero cell lines, with respective standard deviations (*n* = 3) and selectivity.

*Mansoa hirsuta* extracts	LLCMK‐2 cells CC_50_ (μg/mL)	Vero cells CC_50_ (μg/mL)	DENV EC_50_ (μg/mL)	SI	ZIKV EC_50_ (μg/mL)	SI	CHIKV EC_50_ (μg/mL)	SI	MAYV EC_50_ (μg/mL)	SI
Stems	> 500	> 400	NT	—	15.7 ± 1.9	> 25.5	154.5 ± 3.8	> 2.6	101.1 ± 3.2	> 4.0
Leaves	> 500	357.3 ± 2.7	NT	—	34.6 ± 1.7	10.3	86.1 ± 2.8	4.2	86.5 ± 1.9	4.1
Fruits	> 500	> 400	25.4 ± 1.2	> 19.7	28.4 ± 1.9	> 14.1	71.1 ± 1.8	> 5.6	42.3 ± 1.5	> 9.5
Ribavirin (positive control)	NT	370.4 ± 1.2 (1516.7 ± 1.2 μM)	NT	—	94.47 ± 2.70 (386.84 ± 2.7 μM)	3.9	NA	—	29.21 ± 1.4 (119.61 ± 1.39 μM)	12.7
Amantadin (positive control)	NT	84.9 ± 1.2 (561.3 ± 1.2 μM)	NT	—	NT	—	63.37 ± 1.70 (419.0 ± 1.7 μM)	1.34	NT	—
Interferon α 2a (positive control)	NT	—	2.5 ± 10^3^ ^a^, ^b^	—	NT	—	—	—	NT	—

Abbreviations: (—): not determinate; NA: not active; NT: not tested; SI (selectivity index): CC_50 (Vero)_/EC_50 (Vero)_.

^a^ 80%–100% inhibition of cytopathic effect.

^b^ UI/mL concentration.

Thus, *M. hirsuta* extracts demonstrated efficacy comparable to or exceeding standard antivirals. The differential organ‐specific profiles suggest tissue‐specific accumulation of bioactive metabolites, consistent with reported phytochemical variation in Bignoniaceae [7]. The high selectivity indices, particularly stem extract against ZIKV (SI > 25.5) and fruit extract against DENV (SI > 19.7), support further bioassay‐guided fractionation and structure–activity relationship studies using HRMS.

## 4 Conclusion

HRMS enabled the identification of 57 phenolic compounds in *M. hirsuta* EEs, including 40 previously unreported flavonoid conjugates, with mass accuracy consistently below 5 ppm. The diagnostic MS^2^ fragmentation patterns, particularly the base peak at *m/z* 243.1018 [M + H]^+^ (2′,4′,4‐trihydroxydihydrochalcone residue) and neutral loss of 137 Da (tyramine moiety), provided robust structural evidence for characterizing novel oligomeric flavonoids ranging from 380 to 1421 Da. So, five distinct structural classes of dihydrochalcone‐based conjugates were putatively identified: dihydrochalcone‐tyramine, dihydrochalcone‐flavanone (mansoin‐type), dihydrochalcone‐flavan‐3‐ol, dihydrochalcone‐flavone, and dihydrochalcone‐flavonol conjugates. Tissue‐specific distribution patterns were observed, with leaf extracts enriched in Mansoin A–type heterotrimers, stem extracts accumulating phenylethanoid glycosides and cinnamoyl‐mangiferins, and fruit extracts containing simpler dihydrochalcone‐tyramine conjugates. The EEs exhibited low cytotoxicity (CC_50_ > 400–500 μg mL^−1^) and selective antiviral activity against arboviruses. The stem extract demonstrated exceptional selectivity against Zika virus (EC_50_ 15.7 ± 1.9 μg mL^−1^, SI > 25.5), whereas the fruit extract showed the highest potency against dengue virus (EC_50_ 25.4 ± 1.2 μg mL^−1^, SI > 19.7). These selectivity indices substantially exceed the threshold of 10 considered promising for antiviral drug development. Future studies should focus on isolation and NMR spectroscopic confirmation of the novel flavonoid conjugates, particularly the Mansoin‐type heterotrimers and tyramine‐containing oligomers, to definitively establish their interflavonoid connectivity and stereochemistry. Additionally, mechanistic investigations into the antiviral mode of action, including time‐of‐addition assays and molecular docking studies, will elucidate the structure–activity relationships underlying the observed selective inhibition of arboviral replication.

## Funding

This study was supported by Fundação de Amparo à Pesquisa do Estado de Minas Gerais (Grant Nos. APQ‐01375–24, BPD‐00381–22, RED‐00110‐23, and RED‐00170–23), FAPEMIG Research Productivity Scholarship (Grant No. APQ‐06528–24), and National Council for Scientific and Technological Development (CNPq) (Grant No. 402010/2023–0).

## Conflicts of Interest

The authors declare no conflicts of interest.

## Supporting information




**Figure S1:** (A) Conjugate dihydrochalcone and flavanone moieties (Mansoin A Type) (compounds 47, 48, 49, 50, and 51, RT 17.5, 18.1, 18.8, 19.7, and 20.0 min), putatively identified from molecular ion *m/z* 1259.4157 [M + H]^+^ in the ethanolic extract of the *M. hirsuta* **leaves**. (B, B′, B″, B‴, and B′′′′) UV spectra of the compounds 47, 48, 49, 50, and 51, respectively. (C, C′, C″, C‴, and C′′′′) MS^1^ spectra of the compounds 47, 48, 49, 50, and 51, respectively. (D, D′, D″, D‴, and D′′′′) MS^2^ spectra of the compounds 47, 48, 49, 50, and 51, respectively.
**Figure S1:1** (A) Conjugate dihydrochalcone and flavanone moieties (Mansoin A Type) (compounds 47, 48, 49, 50, and 51, RT 17.5, 18.1, 18.8, 19.7, and 20.0 min), putatively identified from molecular ion *m/z* 1259.4148 [M + H]^+^ in the ethanolic extract of the *M. hirsuta* **stems**. (B, B′, and B″) UV spectra of the compounds 47, 48, 49, 50, and 51, respectively. (C, C′, and C″) MS^1^ spectra of the compounds 47, 48, 49, 50, and 51, respectively. (D, D′, and D″) MS^2^ spectra of the compounds 47, 48, 49, 50, and 51, respectively.
**Figure S1:2** (A) Conjugate dihydrochalcone and flavanone moieties (Mansoin A Type) (compounds 47, 48, 49, 50, and 51, RT 17.5, 18.1, 18.8, 19.7, and 20.0 min), putatively identified from molecular ion *m/z* 1259.4196 [M + H]^+^ in the ethanolic extract of the *M. hirsuta* **fruits**. (B, B′, B″, B‴, and B′′′′′) UV spectra of the compounds 47, 48, 49, 50, and 51, respectively. (C, C′, C″, C‴, and C′′′′′) MS^1^ spectra of the compounds 47, 48, 49, 50, and 51, respectively. (D, D′, D″, D‴, and D′′′′′) MS^2^ spectra of the compounds 47, 48, 49, 50, and 51, respectively.
**Figure S2:** (A) Conjugate dihydrochalcone and tyramine moieties (compound 3, RT 12.5 min), putatively identified from molecular ion *m/z* 380.1861 [M + H]^+^ in the ethanolic extract of the *M. hirsuta* **fruits**. (B) UV spectrum. (C) MS^1^ spectrum. (D) MS^2^ spectrum.
**Figure S3:** (A) Proposed fragmentation pathways of ion *m/z* 526.2438 [M + H]^+^ (compounds 7 and 8, RT 13.1 and 13.7 min), putatively identified as a conjugate dihydrochalcone and tyramine moieties in the ethanolic extract of the *M. hirsuta* **fruits**. (B and B′) UV spectra of the compounds 7 and 8, respectively. (C and C′) MS^1^ spectra of the compounds 7 and 8, respectively. (D and D′) MS^2^ spectra of the compounds 7 and 8, respectively.
**Figure S4:** (A) Conjugate dihydrochalcone and tyramine moieties (compounds 9, 10, 11, and 12, RT 11.5, 11.6, 11.9, and 12.2 min), putatively identified from molecular ion *m/z* 542.2395 [M + H]^+^ in the ethanolic extract of the *M. hirsuta* **fruits**. (B, B′, B″, and B‴) UV spectra of the compounds 9, 10, 11, and 12, respectively. (C, C′, C″, and C‴) MS^1^ spectra of the compounds 9, 10, 11, and 12, respectively. (D, D′, D″, and D‴) MS^2^ spectra of the compounds 9, 10, 11, and 12, respectively.
**Figure S4:1** (A) Conjugate dihydrochalcone and tyramine moieties (compounds 9, 10, 11, and 12, RT 11.5, 11.6, 11.9, and 12.2 min), putatively identified from molecular ion *m/z* 542.2372 [M + H]^+^ in the ethanolic extract of the *M. hirsuta* **stems**. (B, B′, B″, and B‴) UV spectra of the compounds 9, 10, 11, and 12, respectively. (C, C′, C″, and C‴) MS^1^ spectra of the compounds 9, 10, 11, and 12, respectively. (D, D′, D″, and D‴) MS^2^ spectra of the compounds 9, 10, 11, and 12, respectively.
**Figure S5:** (A) Proposed fragmentation pathways of the ion *m/z* 946.3863 [M + H]^+^ (compounds 33 and 34, RT 13.9 and 14.4 min, respectively), putatively identified as a conjugate dihydrochalcone and tyramine moieties in the ethanolic extract of the *M. hirsuta* **fruits**. (B and B′) UV spectra of the compounds 33 and 34, respectively. (C and C′) MS^1^ spectra of the compounds 33 and 34, respectively. (D and D′) MS^2^ spectra of the compounds 33 and 34, respectively.
**Figure S6:** (A) Proposed fragmentation pathways of the ion *m/z* 1004.3549 [M + H]^+^ (compounds 36 and 37, RT 14.7 and 14.9 min, respectively), putatively identified as a conjugate dihydrochalcone and flavanone moieties in the ethanolic extract of the *M. hirsuta* **fruits**. (B and B′) UV spectra of the compounds 36 and 37, respectively. (C and C′) MS^1^ spectra of the compounds 36 and 37, respectively. (D and D′) MS^2^ spectra of the compounds 36 and 37, respectively.
**Figure S7:** (A) Proposed fragmentation pathways of ion *m/z* 677.2237 [M + H]^+^ (compound 19, RT 19.2 min), putatively identified as a conjugate dihydrochalcone and flavanone moieties in the ethanolic extract of the *M. hirsuta* **fruits**. (B) UV spectrum. (C) MS^1^ spectrum. (D) MS^2^ spectrum.
**Figure S8:** (A) Proposed fragmentation pathways of ion *m/z* 693.2180 [M + H]^+^ (compound 21, RT 18.1 min), putatively identified as a conjugate dihydrochalcone and flavanonol moieties in the ethanolic extract of the *M. hirsuta* **leaves**. (B) UV spectrum. (C) MS^1^ spectrum. (D) MS^2^ spectrum.
**Figure S8:1** (A) Conjugate dihydrochalcone and flavanonol moieties (compound 21, RT 18.1 min), putatively identified from molecular ion *m/z* 693.2152 [M + H]^+^ in the ethanolic extract of the *M. hirsuta* **stems**. (B and B′) UV spectrum. (C and C′) MS^1^ spectrum. (D and D′) MS^2^ spectrum.
**Figure S8:2** (A) Conjugate dihydrochalcone and flavanonol moieties (compound 21, RT 18.1 min), putatively identified from molecular ion *m/z* 693.2191 [M + H]^+^ in the ethanolic extract of the *M. hirsuta* **fruits**. (B) UV spectrum. (C) MS^1^ spectrum. (D) MS^2^ spectrum.
**Figure S9:** (A) Proposed fragmentation pathways of ion *m/z* 839.2773 [M + H]^+^ (compound 22, RT 15.9 min), putatively identified as a conjugate dihydrochalcone and flavanone moieties in the ethanolic extract of the *M. hirsuta* **fruits**. (B) UV spectrum. (C) MS^1^ spectrum. (D) MS^2^ spectrum.
**Figure S10:** (A) Proposed fragmentation pathways of ion *m/z* 855.2727 [M + H]^+^ (compounds 28 and 29, 16.8 and 17.6 min, respectively), putatively identified as a conjugate dihydrochalcone and flavanone moieties (Mansoin C type) in the ethanolic extract of the *M. hirsuta* **leaves**. (B and B′) UV spectra of the compounds 28 and 29, respectively. (C and C′) MS^1^ spectra of the compounds 28 and 29, respectively. (D and D′) MS^2^ spectra of the compounds 28 and 29, respectively.
**Figure S10:1** (A) Conjugate dihydrochalcone and flavanone moieties (Mansoin C type) (compounds 28 and 29, 16.8 and 17.6 min, respectively), putatively identified from molecular ion *m/z* 855.2683 [M + H]^+^ in the ethanolic extract of the *M. hirsuta* **stems**. (B and B′) UV spectra of the compounds 28 and 29, respectively. (C and C′) MS^1^ spectra of the compounds 28 and 29, respectively. (D and D′) MS^2^ spectra of the compounds 28 and 29, respectively.
**Figure S10:2** (A) Conjugate dihydrochalcone and flavanone moieties (Mansoin C type) (compounds 28 and 29, 16.8 and 17.6 min, respectively), putatively identified from molecular ion *m/z* 855.2727 [M + H]^+^ in the ethanolic extract of the *M. hirsuta* **fruits**. (B and B′) UV spectra of the compounds 28 and 29, respectively. (C and C′) MS^1^ spectra of the compounds 28 and 29, respectively. (D and D′) MS^2^ spectra of the compounds 28 and 29, respectively.
**Figure S11:** (A) Proposed fragmentation pathways of the ion *m/z* 935.3133 [M + H]^+^ (compound 32, RT 15.9 min), putatively identified as a conjugate dihydrochalcone and flavanone moieties in the ethanolic extract of the *M. hirsuta* **fruits**. (B) UV spectrum. (C) MS^1^ spectrum. (D) MS^2^ spectrum.
**Figure S12:** (A) Proposed fragmentation pathways of the ion *m/z* 1097.3680 [M + H]^+^ (compounds 39, 40, 41, and 42, RT 19.3, 19.8, 20.4, and 21.1 min), putatively identified as a conjugate dihydrochalcone and flavanone moieties (Mansoin B type) in the ethanolic extract of the *M. hirsuta* **leaves**. (B, B′, B″, and B‴) UV spectra of the compounds 39, 40, 41, and 42, respectively. (C, C′, C″, and C‴) MS^1^ spectra of the compounds 39, 40, 41, and 42, respectively. (D, D′, D″, and D‴) MS^2^ spectra of the compounds 39, 40, 41, and 42, respectively.
**Figure S12:1** (A) Proposed fragmentation pathways of the ion *m/z* 1097.3613 [M + H]^+^ (compounds 39, 40, 41, and 42, RT 19.3, 19.8, 20.4, and 21.1 min), putatively identified as a conjugate dihydrochalcone and flavanone moieties (Mansoin B type) in the ethanolic extract of the *M. hirsuta* **stems**. (B, B′, B″, and B‴) UV spectra of the compounds 39, 40, 41, and 42, respectively. (C, C′, C″, and C‴) MS^1^ spectra of the compounds 39, 40, 41, and 42, respectively. (D, D′, D″, and D‴) MS^2^ spectra of the compounds 39, 40, 41, and 42, respectively.
**Figure S12:2** (A) Proposed fragmentation pathways of the ion *m/z* 1097.3668 [M + H]^+^ (compounds 39, 40, 41, and 42, RT 19.3, 19.8, 20.4, and 21.1 min), putatively identified as a conjugate dihydrochalcone and flavanone moieties (Mansoin B type) in the ethanolic extract of the *M. hirsuta* **fruits**. (B, B′, B″, and B‴) UV spectra of the compounds 39, 40, 41, and 42, respectively. (C, C′, C″, and C‴) MS^1^ spectra of the compounds 39, 40, 41, and 42, respectively. (D, D′, D″, and D‴) MS^2^ spectra of the compounds 39, 40, 41, and 42, respectively.
**Figure S13:** (A) Proposed fragmentation pathways of the ion *m/z* 1339.4651 [M + H]^+^ (compounds 54 and 55, RT 19.4 and 20.7 min), putatively identified as a conjugate dihydrochalcone and flavanone moieties in the ethanolic extract of the *M. hirsuta* **leaves**. (B and B′) UV spectra of the compounds 54 and 55, respectively. (C and C′) MS^1^ spectra of the compounds 54 and 55, respectively. (D and D′) MS^2^ spectra of the compounds 54 and 55, respectively. *Note:* The scaffold marked with dashed found attached above in the structure putatively identified could be linked in the conjugate dihydrochalcone residue on the left.
**Figure S13:1** (A) Proposed fragmentation pathways of the ion *m/z* 1339.4553 [M + H]^+^ (compounds 54 and 55, RT 19.4 and 20.7 min), putatively identified as a conjugate dihydrochalcone and flavanone moieties in the ethanolic extract of the *M. hirsuta* **stems**. (B) UV spectra of the compounds 54 and 55, respectively. (C) MS^1^ spectra of the compounds 54 and 55, respectively. (D) MS^2^ spectra of the compounds 54 and 55, respectively.
**Figure S14:** (A) Proposed fragmentation pathways of the ion *m/z* 1421.4750 [M + H]^+^ (compounds 56 and 57, RT 17.3 and 17.6 min), putatively identified as a conjugate dihydrochalcone and flavanone moieties in the ethanolic extract of the *M. hirsuta* **fruits**. (B and B′) UV spectra of the compounds 56 and 57, respectively. (C and C′) MS^1^ spectra of the compounds 56 and 57, respectively. (D and D′) MS^2^ spectra of the compounds 56 and 57, respectively. *Note:* The glucose residue marked with dashed found attached above in the structure putatively identified could be linked in the glucose residue of the conjugate dihydrochalcone on the left.
**Figure S15:** (A) Conjugate dihydrochalcone and flavan‐3‐ol moieties (compound 20, RT 19.2 min), putatively identified from molecular ion *m/z* 679.2387 [M + H]^+^ in the ethanolic extract of the *M. hirsuta* **leaves**. (B) UV spectrum. (C) MS^1^ spectrum. (D) MS^2^ spectrum.
**Figure S15:1** (A) Conjugate dihydrochalcone and flavan‐3‐ol moieties (compound 20, RT 18.3 min), putatively identified from molecular ion *m/z* 679.2392 [M + H]^+^ in the ethanolic extract of the *M. hirsuta* **fruits**. (B) UV spectrum. (C) MS^1^ spectrum. (D) MS^2^ spectrum.
**Figure S16:** (A) Proposed fragmentation pathways of ion *m/z* 841.2929 [M + H]^+^ (compound 23, RT 18.5 min), putatively as a conjugate dihydrochalcone and flavan‐3‐ol moieties in the ethanolic extract of the *M. hirsuta* **leaves**. (B) UV spectrum. (C) MS^1^ spectrum. (D) MS^2^ spectrum.
**Figure S17:** (A) Conjugate dihydrochalcone and flavone moieties (compounds 24, 25, 26, and 27, RT 16.1, 16.2, 16.4, and 16.2 min, respectively), putatively identified from molecular ion *m/z* 853.2562 [M + H]^+^ in the ethanolic extract of the *M. hirsuta* **leaves**. (B, B′, B″, and B‴) UV spectra of the compounds 24, 25, 26, and 27, respectively. (C, C′, C″, and C‴) MS^1^ spectra of the compounds 24, 25, 26, and 27, respectively. (D, D′, D″, and D‴) MS^2^ spectra of the compounds 24, 25, 26, and 27, respectively.
**Figure S18:** (A) Proposed fragmentation pathways of the ion *m/z* 999.2938 [M + H]^+^ (compound 35, RT 17.2 min), putatively identified as a conjugate dihydrochalcone and flavone moieties in the ethanolic extract of the *M. hirsuta* **leaves**. (B) UV spectrum. (C) MS^1^ spectrum. (D) MS^2^ spectrum.
**Figure S19:** (A) Proposed fragmentation pathways of the ion *m/z* 1067.3681 [M + H]^+^ (compound 38, RT 220.7 min), putatively identified as a conjugate dihydrochalcone and flavone moieties in the ethanolic extract of the *M. hirsuta* **leaves**. (B) UV spectrum. (C) MS^1^ spectrum. (D) MS^2^ spectrum. *Note:* The scaffold with a neutral loss of 376 Da (blue) found attached to C6 of the flavone A‐ring may be located at C8. The same applies to the neutral loss of 404 Da moiety (orange) attached to C8 of the A‐ring, which may be located at C6.
**Figure S20:** (A) Proposed fragmentation pathways of the ion *m/z* 1257.4069 [M + H]^+^ (compounds 43, 44, 45, and 46, RT 15.9, 16.2, 17.3, and 17.4 min), putatively identified as a conjugate dihydrochalcone and flavone moieties in the ethanolic extract of the *M. hirsuta* **leaves**. (B, B′, B″, and B‴) UV spectra of the compounds 43, 44, 45, and 46, respectively. (C, C′, C″, and C‴) MS^1^ spectra of the compounds 43, 44, 45, and 46, respectively. (D, D′, D″, and D‴) MS^2^ spectra of the compounds 43, 44, 45, and 46, respectively.
**Figure S21:** (A) Conjugate dihydrochalcone and flavonol moieties (compounds 30 and 31, RT 15.5 and 15.9 min, respectively), putatively identified from molecular ion *m/z* 869.2508 [M + H]^+^ in the ethanolic extract of the *M. hirsuta* **leaves**. (B and B′) UV spectra of the compounds 30 and 31, respectively. (C and C′) MS^1^ spectra of the compounds 30 and 31, respectively. (D and D′) MS^2^ spectra of the compounds 30 and 31, respectively.
**Figure S21:1** (A) Conjugate dihydrochalcone and flavonol moieties (compounds 30 and 31, RT 15.5 and 15.9 min, respectively), putatively identified from molecular ion *m/z* 869.2508 [M + H]^+^ in the ethanolic extract of the *M. hirsuta* **stems**. (B and B′) UV spectra of the compounds 30 and 31, respectively. (C and C′) MS^1^ spectra of the compounds 30 and 31, respectively. (D and D′) MS^2^ spectra of the compounds 30 and 31, respectively.
**Figure S22:** (A) Proposed fragmentation pathways of the ion *m/z* 1273.4019 [M + H]^+^ (compounds 52 and 53, RT 15.6 and 15.7 min), putatively identified as a conjugate dihydrochalcone and flavonol moieties in the ethanolic extract of the *M. hirsuta* **leaves**. (B and B′) UV spectra of the compounds 52 and 53, respectively. (C and C′) MS^1^ spectra of the compounds 52 and 53, respectively. (D and D′) MS^2^ spectra of the compounds 52 and 53, respectively.
**Figure S23:** (A) 2′,4′,4‐trihydroxydihydrochalcone (compound 1, RT 14.3 min), putatively identified from molecular ion *m/z* 259.0969 [M + H]^+^ in the ethanolic extract of the *M. hirsuta* **fruits**. (B) UV spectrum. (C) MS^1^ spectrum. (D) MS^2^ spectrum.
**Figure S24:** (A) Eriodictyol (compound 2, RT 15.3 min), putatively identified from molecular ion *m/z* 289.0708 [M + H]^+^ in the ethanolic extract of the *M. hirsuta* **fruits**. (B) UV spectrum. (C) MS^1^ spectrum. (D) MS^2^ spectrum.
**Figure S25:** (A) Apigenin‐7‐*O*‐glucuronide (compound 3, RT 15.7 min), putatively identified from molecular ion *m/z* 447.0919 [M + H]^+^ in the ethanolic extract of the *M. hirsuta* **leaves**. (B) UV spectrum. (C) MS^1^ spectrum. (D) MS^2^ spectrum.
**Figure S26:** (A) Kaempferol‐3‐*O*‐glucoside (compound 5, RT 15.1 min), putatively identified from molecular ion *m/z* 449.1079 [M + H]^+^ in the ethanolic extract of the *M. hirsuta* **leaves**. (B) UV spectrum. (C) MS^1^ spectrum. (D) MS^2^ spectrum.
**Figure S27:** (A) 6‐hydroxyluteolin‐7‐*O*‐glucoside (compound 6, RT 14.0 min), putatively identified from molecular ion *m/z* 465.1023 [M + H]^+^ in the ethanolic extract of the *M. hirsuta* **leaves**. (B) UV spectrum. (C) MS^1^ spectrum. (D) MS^2^ spectrum.
**Figure S28:** (A) 2′‐*O*‐*p*‐hydroxy‐benzoylmangiferin (compound 13, RT 14.4 min), putatively identified from molecular ion *m/z* 543.1110 [M + H]^+^ in the ethanolic extract of the *M. hirsuta* **stems**. (B) UV spectrum. (C) MS^1^ spectrum. (D) MS^2^ spectrum.
**Figure S29:** (A) 2′‐*O*‐*trans*‐cinnamoylmangiferin (compound 14, RT 18.1 min), putatively identified from molecular ion *m/z* 553.1318 [M + H]^+^ in the ethanolic extract of the *M. hirsuta* **stems**. (B) UV spectrum. (C) MS^1^ spectrum. (D) MS^2^ spectrum.
**Figure S30:** (A) 2′‐*O*‐*trans*‐coumaroylmangiferin (compound 15, RT 15.3 min), putatively identified from molecular ion *m/z* 569.1266 [M + H]^+^ in the ethanolic extract of the *M. hirsuta* **stems**. (B) UV spectrum. (C) MS^1^ spectrum. (D) MS^2^ spectrum.
**Figure S31:** (A) 2′‐*O*‐*trans*‐caffeoylmangiferin (compound 16, RT 14.5 min), putatively identified from molecular ion *m/z* 585.1208 [M + H]^+^ in the ethanolic extract of the *M. hirsuta* **stems**. (B) UV spectrum. (C) MS^1^ spectrum. (D) MS^2^ spectrum.
**Figure S32:** (A) Verbascoside (compound 17, RT 14.2 min), putatively identified from molecular ion *m/z* 625.2103 [M + H]^+^ in the ethanolic extract of the *M. hirsuta* **stems**. (B) UV spectrum. (C) MS^1^ spectrum. (D) MS^2^ spectrum.
**Figure S33:** (A) Isoverbascoside (compound 18, RT 14.7 min), putatively identified from molecular ion *m/z* 625.2101 [M + H]^+^ in the ethanolic extract of the *M. hirsuta* **stems**. (B) UV spectrum. (C) MS^1^ spectrum. (D) MS^2^ spectrum.

## Data Availability

The data that support the findings of this study are available in the Supporting Information of this article.

## References

[1] J. Ma , K. Li , S. Shi , J. Li , S. Tang , and L. Liu , “The Application of UHPLC‐HRMS for Quality Control of Traditional Chinese Medicine,” Frontiers in Pharmacology 13 (2022): 922488. 10.3389/fphar.2022.922488 PMC9201421 35721122

[2] A. Rivera‐Pérez , P. García‐Pérez , R. Romero‐González , A. Garrido Frenich , and L. Lucini , “UHPLC‐QTOF‐HRMS Metabolomics Insight on the Origin and Processing Authentication of Thyme by Comprehensive Fingerprinting and Chemometrics,” Food Chemistry 407 (2023): 135123. 10.1016/j.foodchem.2022.135123 36493482

[3] J. Hubert , J.‐M. Nuzillard , and J.‐H. Renault , “Dereplication Strategies in Natural Product Research: Tools and Methodologies Behind the Same Concept,” Phytochemistry Reviews 16 (2017): 55–95.

[4] P. M. Allard , T. Péresse , J. Bisson , et al., “Integration of Molecular Networking and In Silico MS/MS Fragmentation for Natural Products Dereplication,” Analytical Chemistry 88 (2016): 3317–3323. 10.1021/acs.analchem.5b04804 26882108

[5] H. Horai , M. Arita , S. Kanaya , et al., “MassBank: A Public Repository for Sharing Mass Spectral Data for Life Sciences,” Journal of Mass Spectrometry 45 (2010): 703–714. 10.1002/jms.1777 20623627

[6] I. O. Pereira , R. F. Queiroz , E. A. Siqueira , et al., “Phytochemical Investigation and Biological Activities of *Mansoa hirsuta* ,” Anais da Academia Brasileira de Ciências 89 (2017): 317–331. 10.1590/0001-3765201720160585 28423086

[7] M. S. Alves , et al., “Ethnopharmacological Uses of Bignoniaceae Species in Brazil,” Biophysica 3 (2023): 425–445.

[8] P. R. V. Campana , C. M. Coleman , M. M. Teixeira , D. Ferreira , and F. C. Braga , “TNF‐α Inhibition Elicited by Mansoins A and B, Heterotrimeric Flavonoids Isolated From *Mansoa hirsuta* ,” Journal of Natural Products 77 (2014): 824–830. 10.1021/np400929g 24576254

[9] P. R. V. Campana , C. M. Coleman , L. P. Sousa , M. M. Teixeira , D. Ferreira , and F. C. Braga , “Mansoin F: TNF‐α Inhibiting Heterotrimeric Flavonoid From *Mansoa hirsuta* DC,” Planta Medica 82 (2016): C22.

[10] L. T. Lin , W. C. Hsu , and C. C. Lin , “Antiviral Natural Products and Herbal Medicines,” Journal of Traditional and Complementary Medicine 4 (2014): 24–35. 10.4103/2225-4110.124335 PMC4032839 24872930

[11] H. Zakaryan , E. Arabyan , A. Oo , and K. Zandi , “Flavonoids: Promising Natural Compounds Against Viral Infections,” Archives of Virology 162 (2017): 2539–2551. 10.1007/s00705-017-3417-y PMC7087220 28547385

[12] S. Jo , S. Kim , D. H. Shin , and M. S. Kim , “Inhibition of SARS‐CoV Protease by Flavonoids,” Journal of Enzyme Inhibition and Medicinal Chemistry 35 (2020): 145–151. 10.1080/14756366.2019.1690480 PMC6882434 31724441

[13] A. L. Harvey , R. Edrada‐Ebel , and R. J. Quinn , “The Re‐Emergence of Natural Products for Drug Discovery,” Nature Reviews. Drug Discovery 14 (2015): 111–129. 10.1038/nrd4510 25614221

[14] A. F. G. Cruz , A. C. C. Reis , J. A. C. Sousa , et al., “High‐Resolution Mass Spectrometry Identification and Characterization of Flavonoids From *Fridericia chica* Leaves Extract With Anti‐Arbovirus Activity,” Molecules 27 (2022): 6043. 10.3390/molecules27186043 PMC9501042 36144777

[15] A. C. C. Reis , B. M. Silva , H. M. Moura , G. R. Pereira , and G. C. Brandão , “Anti‐Zika Virus Activity and Chemical Characterization by UPLC‐DAD‐UV‐MS of Ethanol Extracts in *Tecoma* Species,” BMC Complementary Medicine and Therapies 20 (2020): 246. 10.1186/s12906-020-03040-0 PMC7412813 32767975

[16] A. C. C. Reis , G. M. Valente , B. M. Silva , C. L. B. Magalhães , M. Kohlhoff , and G. C. Brandão , “Anti‐Arboviral Activity and Chemical Characterization of Hispidulin and Ethanolic Extracts From *Millingtonia hortensis* lf and *Oroxylum indicum* (L.) Kurz (Bignoniaceae),” Natural Product Research 37 (2023): 613–617. 10.1080/14786419.2022.2065485 35428404

[17] T. Mosmann , “Rapid Colorimetric Assay for Cellular Growth and Survival: Application to Proliferation and Cytotoxicity Assays,” Journal of Immunological Methods 65 (1983): 55–63. 10.1016/0022-1759(83)90303-4 6606682

[18] G. C. Brandão , E. G. Kroon , J. D. Souza Filho , and A. B. Oliveira , “Antiviral Activity of *Fridericia formosa* (Bureau) L.G. Lohmann (Bignoniaceae) Extracts and Constituents,” Journal of Tropical Medicine 2017 (2017): 1–11. 10.1155/2017/6106959 PMC5467316 28634494

[19] P. Cos , A. J. Vlietinck , D. Vanden Berghe , and L. Maes , “Anti‐Infective Potential of Natural Products: How to Develop a Stronger In Vitro Proof‐Of‐Concept,” Journal of Ethnopharmacology 106 (2006): 290–302. 10.1016/j.jep.2006.04.003 16698208

[20] M. Irakli , A. Skendi , E. Bouloumpasi , P. Chatzopoulou , and C. G. Biliaderis , “LC‐MS Identification and Quantification of Phenolic Compounds in Solid Residues From the Essential Oil Industry,” Antioxidants 10 (2021): 2016. 10.3390/antiox10122016 PMC8698398 34943119

[21] A. Evidente and M. Mais , “Natural Bioactive Cinnamoyltyramine Alkylamides and Co‐Metabolites,” Biomolecules 11 (2021): 1765. 10.3390/biom11121765 PMC8698393 34944409

[22] F. Tureček and F. W. McLafferty , Interpretation of Mass Spectra (University Science Books, 1993), 37–38.

[23] D. O. Sparkman , Mass Spectrometry Desk Reference (Global View Publishing, 2007), 64.

[24] G. Y. Sim , S.‐M. Yang , B. G. Kim , and J.‐H. Ahn , “Bacterial Synthesis of *N*‐Hydroxycinnamoyl Phenethylamines and Tyramines,” Microbial Cell Factories 14 (2015): 162. 10.1186/s12934-015-0353-y PMC4603808 26463041

[25] B. Portet , N. Fabre , R. Rozenberg , J.‐L. Habib‐Jiwan , C. Moulis , and J. Quetin‐Leclercq , “Analysis of Minor Flavonoids in *Piper hostmannianum* var. *berbicense* Using Liquid Chromatography Coupled With Atmospheric Pressure Chemical Ionization Mass Spectrometry,” Journal of Chromatography. A 1210 (2008): 45–54. 10.1016/j.chroma.2008.09.055 18838145

[26] W. Feng , Z. Hao , and M. Li , “Isolation and Structure Identification of Flavonoids. In: Flavonoids—From Biosynthesis to Human Health,” in Flavonoids—From Biosynthesis to Human Health, ed. G. C. Justino (InTech, 2017), 17–43.

[27] H. Enomoto , S. Takahashi , S. Takeda , and H. H. Hatta , “Distribution of Flavan‐3‐Ol Species in Ripe Strawberry Fruit Revealed by Matrix‐Assisted Laser Desorption/Ionization‐Mass Spectrometry Imaging,” Molecules 25 (2020): 103. 10.3390/molecules25010103 PMC6982903 31888096

[28] S. Yuzuak , J. Ballington , and D.‐Y. Xie , “HPLC‐qTOF‐MS/MS‐Based Profiling of Flavan‐3‐ols and Dimeric Proanthocyanidins in Berries of Two Muscadine Grape Hybrids FLH 13‐11 and FLH 17‐66,” Metabolites 8 (2018): 57. 10.3390/metabo8040057 PMC6316709 30261603

[29] D. Tsimogiannis , M. Samiotaki , G. Panayotou , and V. Oreopoulou , “Characterization of Flavonoid Subgroups and Hydroxy Substitution by HPLC‐MS/MS,” Molecules 12 (2007): 593–606. 10.3390/12030593 PMC6149353 17851414

[30] C. Jiang and P. J. Gates , “Systematic Characterisation of the Fragmentation of Flavonoids Using High‐Resolution Accurate Mass Electrospray Tandem Mass Spectrometry,” Molecules 29 (2024): 5246. 10.3390/molecules29225246 PMC11596855 39598635

[31] Y. Liu , J. Qian , J. Li , et al., “Hydroxylation Decoration Patterns of Flavonoids in Horticultural Crops: Chemistry, Bioactivity, and Biosynthesis,” Horticulture Research 9 (2022): uhab068. 10.1093/hr/uhab068 PMC8945325 35048127

[32] S. Kumar and A. K. Pandey , “Chemistry and Biological Activities of Flavonoids: An Overview,” Scientific World Journal 2013 (2013): 162750. 10.1155/2013/162750 PMC3891543 24470791

[33] J. M. Fonseca , A. C. C. Reis , G. R. Pereira , et al., “Chromatographic Profile of Xanthones and Flavonoids in the Anti‐Dengue Extracts of *Fridericia samydoides* (Cham.) L.G. Lohmann (Bignoniaceae),” Brazilian Journal of Pharmaceutical Sciences 58 (2022): e18802.

[34] S. Tammar , N. Salem , W. A. Wannes , et al., “Chemometric Profiling and Bioactivity of Verbena (*Aloysia citrodora*) Methanolic Extract From Four Localities in Tunisia,” Food 10 (2021): 2912. 10.3390/foods10122912 PMC8700817 34945462

[35] T. Du , Y. Wang , H. Xie , D. Liang , and S. Gao , “Fragmentation Patterns of Phenolic C‐Glycosides in Mass Spectrometry Analysis,” Molecules 29 (2024): 2953. 10.3390/molecules29132953 PMC11243344 38998905

[36] Q. Chen , S. Xue , F. Jia , et al., “Systematic Analysis of Chemical Constituents and Pharmacokinetics of Baihe Dihuang Decoction In Vivo by UHPLC‐MS/MS,” Journal of Separation Science 48 (2025): e70281. 10.1002/jssc.70281 40994161

