# Supplementary material for: UHPLC‐DAD‐HRMS2 Characterization of Novel Dihydrochalcone Conjugates From the Antiviral Mansoa hirsuta Extracts: Diagnostic Fragmentation Pathways and Dereplication of Oligomeric Polyphenols
Source: J Mass Spectrom. 2026 Nov 1;61(11):e70128. doi: 10.1002/jms.70128 (PMC13725079; doi:10.1002/jms.70128)
Supplement: Supplementary file 1 [file JMS-61-e70128-s001.docx]

**UHPLC-DAD-HRMS^2^ characterization of novel dihydrochalcone conjugates from the antiviral *Mansoa hirsuta* extracts: diagnostic fragmentation pathways and dereplication of oligomeric polyphenols**

***UHPLC-HRMS*^2^ *characterization of novel* dihydrochalcone *conjugates in Mansoa hirsuta***

Adriana Cotta Cardoso Reis^1^, Breno de Mello Silva^2^, Cíntia Lopes de Brito Magalhães^2^, Erna G. Kroon^3^, Douglas da Costa Gontijo^4^, Markus Kohlhoff^5^, and Geraldo Célio Brandão^1*^

^1^Programa de Pós-graduação em Ciências Farmacêuticas, Escola de Farmácia, Universidade Federal de Ouro Preto, Campus Morro do Cruzeiro, Ouro Preto Zip code 35.402-163, Minas Gerais, Brazil;

^2^Departamento de Ciências Biológicas, ICEB, Universidade Federal de Ouro Preto, Campus Morro do Cruzeiro, Ouro Preto Zip code 35.402-163, MG, Brazil;

^3^Instituto de Ciências Biológicos, Universidade Federal de Minas Gerais, Av. Antônio Carlos, 6627, Belo Horizonte Zip code 31.270-901, Minas Gerais, Brazil.

^4^Divisão de Química Orgânica, Instituto de Química, Universidade de Brasília, Brasília Zip code 70.910-900, Distrito Federal, Brazil.

^5^Laboratório de Química de Produtos Naturais Bioativos, Fundação Oswaldo Cruz. Instituto René Rachou, Belo Horizonte Zip code 30.190-009, Minas Gerais, Brazil.

*Correspondence: celiobrandao@ufop.edu.br; Tel.: +55-31-3559-1088; Fax: +55-31-3559-1069

**Figure S1.** A) Conjugate dihydrochalcone and flavanone moieties (Mansoin A Type) (compounds 47, 48, 49, 50 and 51, RT 17.5, 18.1, 18.8, 19.7 and 20.0 min), putatively identified from molecular ion *m/z* 1259.4157 [M + H]^+^ in the ethanolic extract of the *M. hirsuta* **leaves**. B, B’, B’’, B’’’ and B’’’’) UV spectra of the compounds 47, 48, 49, 50 and 51, respectively. C, C’, C’’, C’’’ and C’’’’) MS^1^ spectra of the compounds 47, 48, 49, 50 and 51, respectively. D, D’, D’’, D’’’ and D’’’’) MS^2^ spectra of the compounds 47, 48, 49, 50 and 51, respectively.

A

B

C

D

B’

C’

D’

B’’

C’’

D’’

B’’’

C’’’

D’’’

B’’’’

C’’’’

D’’’’

**Figure S1.1.** A) Conjugate dihydrochalcone and flavanone moieties (Mansoin A Type) (compounds 47, 48, 49, 50 and 51, RT 17.5, 18.1, 18.8, 19.7 and 20.0 min), putatively identified from molecular ion *m/z* 1259.4148 [M + H]^+^ in the ethanolic extract of the *M. hirsuta* **stems**. B, B’, and B’’) UV spectra of the compounds 47, 48, 49, 50 and 51, respectively. C, C’, and C’’) MS^1^ spectra of the compounds 47, 48, 49, 50 and 51, respectively. D, D’, and D’’) MS^2^ spectra of the compounds 47, 48, 49, 50 and 51, respectively.

A

B

C

D

B’

C’

D’

B’’

C’’

D’’

**Figure S1.2.** A) Conjugate dihydrochalcone and flavanone moieties (Mansoin A Type) (compounds 47, 48, 49, 50 and 51, RT 17.5, 18.1, 18.8, 19.7 and 20.0 min), putatively identified from molecular ion *m/z* 1259.4196 [M + H]^+^ in the ethanolic extract of the *M. hirsuta* **fruits**. B, B’, B’’, B’’’ and B’’’’) UV spectra of the compounds 47, 48, 49, 50 and 51, respectively. C, C’, C’’, C’’’ and C’’’’) MS^1^ spectra of the compounds 47, 48, 49, 50 and 51, respectively. D, D’, D’’, D’’’ and D’’’’) MS^2^ spectra of the compounds 47, 48, 49, 50 and 51, respectively.

A

B

C

D

B’

C’

D’

B’’

C’’

D’’

B’’’

C’’’

D’’’

B’’’’

C’’’’

D’’’’

**Figure S2.** A) Conjugate dihydrochalcone and tyramine moieties (compound 3, RT 12.5 min), putatively identified from molecular ion *m/z* 380.1861 [M + H]^+^ in the ethanolic extract of the *M. hirsuta* **fruits**. B) UV spectrum. C) MS^1^ spectrum. D) MS^2^ spectrum.

A

B

C

D

**Figure S3.** A) Proposed fragmentation pathways of ion *m/z* 526.2438 [M + H]^+^ (compounds 7 and 8, RT 13.1 and 13.7 min), putatively identified as a conjugate dihydrochalcone and tyramine moieties in the ethanolic extract of the *M. hirsuta* **fruits**. B and B’) UV spectra of the compounds 7 and 8, respectively. C and C’) MS^1^ spectra of the compounds 7 and 8, respectively. D and D’) MS^2^ spectra of the compounds 7 and 8, respectively.

A

B

C

D

B’

C’

D’

**Figure S4.** A) Conjugate dihydrochalcone and tyramine moieties (compounds 9, 10, 11 and 12, RT 11.5, 11.6, 11.9 and 12.2 min), putatively identified from molecular ion *m/z* 542.2395 [M + H]^+^ in the ethanolic extract of the *M. hirsuta* **fruits**. B, B’, B’’ and B’’’) UV spectra of the compounds 9, 10, 11 and 12, respectively. C, C’, C’’ and C’’’) MS^1^ spectra of the compounds 9, 10, 11 and 12, respectively. D, D’, D’’ and D’’’) MS^2^ spectra of the compounds 9, 10, 11 and 12, respectively.

A

B

B’

C

D

B’

C’

D’

B’’

C’’

D’’

B’’’

C’’’

D’’’

**Figure S4.1.** A) Conjugate dihydrochalcone and tyramine moieties (compounds 9, 10, 11 and 12, RT 11.5, 11.6, 11.9 and 12.2 min), putatively identified from molecular ion *m/z* 542.2372 [M + H]^+^ in the ethanolic extract of the *M. hirsuta* **stems**. B, B’, B’’ and B’’’) UV spectra of the compounds 9, 10, 11 and 12, respectively. C, C’, C’’ and C’’’) MS^1^ spectra of the compounds 9, 10, 11 and 12, respectively. D, D’, D’’ and D’’’) MS^2^ spectra of the compounds 9, 10, 11 and 12, respectively.

A

**B**

C

D

B’

C’

D’

B’’

C’’

D’’

B’’’

C’’’

D’’’

**Figure S5.** A) Proposed fragmentation pathways of the ion *m/z* 946.3863 [M + H]^+^ (compounds 33 and 34, RT 13.9 and 14.4 min, respectively), putatively identified as a conjugate dihydrochalcone and tyramine moieties in the ethanolic extract of the *M. hirsuta* **fruits**. B and B’) UV spectra of the compounds 33 and 34, respectively. C and C’) MS^1^ spectra of the compounds 33 and 34, respectively. D and D’) MS^2^ spectra of the compounds 33 and 34, respectively.

A

B

C

D

B’

C’

D’

**Figure S6.** A) Proposed fragmentation pathways of the ion *m/z* 1004.3549 [M + H]^+^ (compounds 36 and 37, RT 14.7 and 14.9 min, respectively), putatively identified as a conjugate dihydrochalcone and flavanone moieties in the ethanolic extract of the *M. hirsuta* **fruits**. B and B’) UV spectra of the compounds 36 and 37, respectively. C and C’) MS^1^ spectra of the compounds 36 and 37, respectively. D and D’) MS^2^ spectra of the compounds 36 and 37, respectively.

A

B

C

D

B’

C’

D’

**Figure S7.** A) Proposed fragmentation pathways of ion *m/z* 677.2237 [M + H]^+^ (compound 19, RT 19.2 min), putatively identified as a conjugate dihydrochalcone and flavanone moieties in the ethanolic extract of the *M. hirsuta* **fruits**. B) UV spectrum. C) MS^1^ spectrum. D) MS^2^ spectrum.

A

B

C

D

**Figure S8.** A) Proposed fragmentation pathways of ion *m/z* 693.2180 [M + H]^+^ (compound 21, RT 18.1 min), putatively identified as a conjugate dihydrochalcone and flavanonol moieties in the ethanolic extract of the *M. hirsuta* **leaves**. B) UV spectrum. C) MS^1^ spectrum. D) MS^2^ spectrum.

A

B

C

D

**Figure S8.1.** A) Conjugate dihydrochalcone and flavanonol moieties (compound 21, RT 18.1 min), putatively identified from molecular ion *m/z* 693.2152 [M + H]^+^ in the ethanolic extract of the *M. hirsuta* **stems**. B and B’) UV spectrum. C and C’) MS^1^ spectrum. D and D’) MS^2^ spectrum.

A

B

C

D

**Figure S8.2.** A) Conjugate dihydrochalcone and flavanonol moieties (compound 21, RT 18.1 min), putatively identified from molecular ion *m/z* 693.2191 [M + H]^+^ in the ethanolic extract of the *M. hirsuta* **fruits**. B) UV spectrum. C) MS^1^ spectrum. D) MS^2^ spectrum.

A

B

C

D

**Figure S9.** A) Proposed fragmentation pathways of ion *m/z* 839.2773 [M + H]^+^ (compound 22, RT 15.9 min), putatively identified as a conjugate dihydrochalcone and flavanone moieties in the ethanolic extract of the *M. hirsuta* **fruits**. B) UV spectrum. C) MS^1^ spectrum. D) MS^2^ spectrum.

A

B

C

D

**Figure S10.** A) Proposed fragmentation pathways of ion *m/z* 855.2727 [M + H]^+^ (compounds 28 and 29, 16.8 and 17.6 min, respectively), putatively identified as a conjugate dihydrochalcone and flavanone moieties (Mansoin C type) in the ethanolic extract of the *M. hirsuta* **leaves**. B and B’) UV spectra of the compounds 28 and 29, respectively. C and C’) MS^1^ spectra of the compounds 28 and 29, respectively. D and D’) MS^2^ spectra of the compounds 28 and 29, respectively.

A

B

C

D

B’

C’

D’

**Figure S10.1.** A) Conjugate dihydrochalcone and flavanone moieties (Mansoin C type) (compounds 28 and 29, 16.8 and 17.6 min, respectively), putatively identified from molecular ion *m/z* 855.2683 [M + H]^+^ in the ethanolic extract of the *M. hirsuta* **stems**. B and B’) UV spectra of the compounds 28 and 29, respectively. C and C’) MS^1^ spectra of the compounds 28 and 29, respectively. D and D’) MS^2^ spectra of the compounds 28 and 29, respectively.

A

B

C

D

B’

C’

D’

**Figure S10.2.** A) Conjugate dihydrochalcone and flavanone moieties (Mansoin C type) (compounds 28 and 29, 16.8 and 17.6 min, respectively), putatively identified from molecular ion *m/z* 855.2727 [M + H]^+^ in the ethanolic extract of the *M. hirsuta* **fruits**. B and B’) UV spectra of the compounds 28 and 29, respectively. C and C’) MS^1^ spectra of the compounds 28 and 29, respectively. D and D’) MS^2^ spectra of the compounds 28 and 29, respectively.

C

B

D

C’

B’

D’

**Figure S11.** A) Proposed fragmentation pathways of the ion *m/z* 935.3133 [M + H]^+^, (compound 32, RT 15.9 min), putatively identified as a conjugate dihydrochalcone and flavanone moieties in the ethanolic extract of the *M. hirsuta* **fruits**. B) UV spectrum. C) MS^1^ spectrum. D) MS^2^ spectrum.

A

B

C

D

**Figure S12.** A) Proposed fragmentation pathways of the ion *m/z* 1097.3680 [M + H]^+^ (compounds 39, 40, 41 and 42, RT 19.3, 19.8, 20.4 and 21.1 min), putatively identified as a conjugate dihydrochalcone and flavanone moieties (Mansoin B type) in the ethanolic extract of the *M. hirsuta* **leaves**. B, B’, B’’ and B’’’) UV spectra of the compounds 39, 40, 41 and 42, respectively. C, C’, C’’ and C’’’) MS^1^ spectra of the compounds 39, 40, 41 and 42, respectively. D, D’, D’’ and D’’’) MS^2^ spectra of the compounds 39, 40, 41 and 42, respectively.

A

B

C

B’

C’

D’

B’’

C’’

D’’

B’’’

C’’’

D’’’

**F****igure S12.1.** A) Proposed fragmentation pathways of the ion *m/z* 1097.3613 [M + H]^+^ (compounds 39, 40, 41 and 42, RT 19.3, 19.8, 20.4 and 21.1 min), putatively identified as a conjugate dihydrochalcone and flavanone moieties (Mansoin B type) in the ethanolic extract of the *M. hirsuta* **stems**. B, B’, B’’ and B’’’) UV spectra of the compounds 39, 40, 41 and 42, respectively. C, C’, C’’ and C’’’) MS^1^ spectra of the compounds 39, 40, 41 and 42, respectively. D, D’, D’’ and D’’’) MS^2^ spectra of the compounds 39, 40, 41 and 42, respectively.

A

B

C

D

B’

C’

D’

B’’

C’’

D’’

B’’’

C’’’

D’’’

**Figure S12.2.** A) Proposed fragmentation pathways of the ion *m/z* 1097.3668 [M + H]^+^ (compounds 39, 40, 41 and 42, RT 19.3, 19.8, 20.4 and 21.1 min), putatively identified as a conjugate dihydrochalcone and flavanone moieties (Mansoin B type) in the ethanolic extract of the *M. hirsuta* **fruits**. B, B’, B’’ and B’’’) UV spectra of the compounds 39, 40, 41 and 42, respectively. C, C’, C’’ and C’’’) MS^1^ spectra of the compounds 39, 40, 41 and 42, respectively. D, D’, D’’ and D’’’) MS^2^ spectra of the compounds 39, 40, 41 and 42, respectively.

A

B

C

D

B’

C’

D’

B’’

C’’

D’’

B’’’

C’’’

D’’’

**Figure S13.** A) Proposed fragmentation pathways of the ion *m/z* 1339.4651 [M + H]^+^ (compounds 54 and 55, RT 19.4 and 20.7 min), putatively identified as a conjugate dihydrochalcone and flavanone moieties in the ethanolic extract of the *M. hirsuta* **leaves**. B and B’) UV spectra of the compounds 54 and 55, respectively. C and C’) MS^1^ spectra of the compounds 54 and 55, respectively. D and D’) MS^2^ spectra of the compounds 54 and 55, respectively. Note: The scaffold marked with dashed found attached above in the structure putatively identified could be linked in the conjugate dihydrochalcone residue on the left.

A

B

C

D

B’

C’

D’

**Figure S13.1.** A) Proposed fragmentation pathways of the ion *m/z* 1339.4553 [M + H]^+^ (compounds 54 and 55, RT 19.4 and 20.7 min), putatively identified as a conjugate dihydrochalcone and flavanone moieties in the ethanolic extract of the *M. hirsuta* **stems**. B) UV spectra of the compounds 54 and 55, respectively. C) MS^1^ spectra of the compounds 54 and 55, respectively. D) MS^2^ spectra of the compounds 54 and 55, respectively.

A

B

C

D

**Figure S14.** A) Proposed fragmentation pathways of the ion *m/z* 1421.4750 [M + H]^+^ (compounds 56 and 57, RT 17.3 and 17.6 min), putatively identified as a conjugate dihydrochalcone and flavanone moieties in the ethanolic extract of the *M. hirsuta* **fruits**. B and B’) UV spectra of the compounds 56 and 57, respectively. C and C’) MS^1^ spectra of the compounds 56 and 57, respectively. D and D’) MS^2^ spectra of the compounds 56 and 57, respectively. Note: The glucose residue marked with dashed found attached above in the structure putatively identified could be linked in the glucose residue of the conjugate dihydrochalcone on the left.

A

B

C

D

B’

C’

D’

**Figure S15.** A) Conjugate dihydrochalcone and flavan-3-ol moieties (compound 20, RT 19.2 min), putatively identified from molecular ion *m/z* 679.2387 [M + H]^+^ in the ethanolic extract of the *M. hirsuta* **leaves**. B) UV spectrum. C) MS^1^ spectrum. D) MS^2^ spectrum.

A

B

C

D

**Figure S15.1.** A) Conjugate dihydrochalcone and flavan-3-ol moieties (compound 20, RT 18.3 min), putatively identified from molecular ion *m/z* 679.2392 [M + H]^+^ in the ethanolic extract of the *M. hirsuta* **fruits**. B) UV spectrum. C) MS^1^ spectrum. D) MS^2^ spectrum.

**A**

C

B

D

**Figure S16.** A) Proposed fragmentation pathways of ion *m/z* 841.2929 [M + H]^+^ (compound 23, RT 18.5 min), putatively as a conjugate dihydrochalcone and flavan-3-ol moieties in the ethanolic extract of the *M. hirsuta* **leaves**. B) UV spectrum. C) MS^1^ spectrum. D) MS^2^ spectrum.

A

B

C

D

**Figure S17.** A) Conjugate dihydrochalcone and flavone moieties (compounds 24, 25, 26 and 27, RT 16.1, 16.2, 16.4 and 16.2 min, respectively), putatively identified from molecular ion *m/z* 853.2562 [M + H]^+^ in the ethanolic extract of the *M. hirsuta* **leaves**. B, B’, B’’ and B’’’) UV spectra of the compounds 24, 25, 26 and 27, respectively. C, C’, C’’ and C’’’) MS^1^ spectra of the compounds 24, 25, 26 and 27, respectively. D, D’, D’’ and D’’’) MS^2^ spectra of the compounds 24, 25, 26 and 27, respectively.

A

B

C

D

B’

C’

D’

B’’

C’’

D’’

B’’’

C’’’

D’’’

**Figure S18.** A) Proposed fragmentation pathways of the ion *m/z* 999.2938 [M + H]^+^ (compound 35, RT 17.2 min), putatively identified as a conjugate dihydrochalcone and flavone moieties in the ethanolic extract of the *M. hirsuta* **leaves**. B) UV spectrum. C) MS^1^ spectrum. D) MS^2^ spectrum.

A

B

C

D

**Figure S19.** A) Proposed fragmentation pathways of the ion *m/z* 1067.3681 [M + H]^+^ (compound 38, RT 220.7 min), putatively identified as a conjugate dihydrochalcone and flavone moieties in the ethanolic extract of the *M. hirsuta* **leaves**. B) UV spectrum. C) MS^1^ spectrum. D) MS^2^ spectrum. Note: The scaffold with a neutral loss of 376 Da (blue) found attached to C6 of the flavone A-ring may be located at C8. The same applies to the neutral loss of 404 Da moiety (orange) attached to C8 of the A-ring, which may be located at C6.

A

B

C

D

**Figure S20.** A) Proposed fragmentation pathways of the ion *m/z* 1257.4069 [M + H]^+^ (compounds 43, 44, 45 and 46, RT 15.9, 16.2, 17.3 and 17.4 min), putatively identified as a conjugate dihydrochalcone and flavone moieties in the ethanolic extract of the *M. hirsuta* **leaves**. B, B’, B’’ and B’’’) UV spectra of the compounds 43, 44, 45 and 46, respectively. C, C’, C’’ and C’’’) MS^1^ spectra of the compounds 43, 44, 45 and 46, respectively. D, D’, D’’ and D’’’) MS^2^ spectra of the compounds 43, 44, 45 and 46, respectively.

A

B

C

D

B’

C’

D’

B’’

C’’

D’’

B’’’

C’’’

D’’’

**Figure S21.** A) Conjugate dihydrochalcone and flavonol moieties (compounds 30 and 31, RT 15.5 and 15.9 min, respectively), putatively identified from molecular ion *m/z* 869.2508 [M + H]^+^ in the ethanolic extract of the *M. hirsuta* **leaves**. B and B’) UV spectra of the compounds 30 and 31, respectively. C and C’) MS^1^ spectra of the compounds 30 and 31, respectively. D and D’) MS^2^ spectra of the compounds 30 and 31, respectively.

A

B

C

D

B’

C’

D’

**Figure S21.1.** A) Conjugate dihydrochalcone and flavonol moieties (compounds 30 and 31, RT 15.5 and 15.9 min, respectively), putatively identified from molecular ion *m/z* 869.2508 [M + H]^+^ in the ethanolic extract of the *M. hirsuta* **stems**. B and B’) UV spectra of the compounds 30 and 31, respectively. C and C’) MS^1^ spectra of the compounds 30 and 31, respectively. D and D’) MS^2^ spectra of the compounds 30 and 31, respectively.

A

B

C

D

B’

C’

D’

**Figure S22.** A) Proposed fragmentation pathways of the ion *m/z* 1273.4019 [M + H]^+^ (compounds 52 and 53, RT 15.6 and 15.7 min), putatively identified as a conjugate dihydrochalcone and flavonol moieties in the ethanolic extract of the *M. hirsuta* **leaves**. B and B’) UV spectra of the compounds 52 and 53, respectively. C and C’) MS^1^ spectra of the compounds 52 and 53, respectively. D and D’) MS^2^ spectra of the compounds 52 and 53, respectively.

A

B

C

D

B’

C’

D’

**Figure S23.** A) 2',4',4-trihydroxydihydrochalcone (compound 1, RT 14.3 min), putatively identified from molecular ion *m/z* 259.0969 [M + H]^+^ in the ethanolic extract of the *M. hirsuta* **fruits**. B) UV spectrum. C) MS^1^ spectrum. D) MS^2^ spectrum.

A

B

C

D

**Figure S24.** A) Eriodictyol (compound 2, RT 15.3 min), putatively identified from molecular ion *m/z* 289.0708 [M + H]^+^ in the ethanolic extract of the *M. hirsuta* **fruits**. B) UV spectrum. C) MS^1^ spectrum. D) MS^2^ spectrum.

A

B

C

D

**Figure S25.** A) Apigenin-7-*O*-glucuronide (compound 3, RT 15.7 min), putatively identified from molecular ion *m/z* 447.0919 [M + H]^+^ in the ethanolic extract of the *M. hirsuta* **leaves**. B) UV spectrum. C) MS^1^ spectrum. D) MS^2^ spectrum.

A

C

B

D

**Figure S26.** A) Kaempferol-3-*O*-glucoside (compound 5, RT 15.1 min), putatively identified from molecular ion *m/z* 449.1079 [M + H]^+^ in the ethanolic extract of the *M. hirsuta* **leaves**. B) UV spectrum. C) MS^1^ spectrum. D) MS^2^ spectrum.

A

B

C

D

**Figure S27.** A) 6-hydroxyluteolin-7-*O*-glucoside (compound 6, RT 14.0 min), putatively identified from molecular ion *m/z* 465.1023 [M + H]^+^ in the ethanolic extract of the *M. hirsuta* **leaves**. B) UV spectrum. C) MS^1^ spectrum. D) MS^2^ spectrum.

A

B

C

D

**Figure S28.** A) *2′-O-p*-hydroxy-benzoylmangiferin (compound 13, RT 14.4 min), putatively identified from molecular ion *m/z* 543.1110 [M + H]^+^ in the ethanolic extract of the *M. hirsuta* **stems**. B) UV spectrum. C) MS^1^ spectrum. D) MS^2^ spectrum.

B

A

C

D

**Figure S29.** A) *2′-O-trans*-cinnamoylmangiferin (compound 14, RT 18.1 min), putatively identified from molecular ion *m/z* 553.1318 [M + H]^+^ in the ethanolic extract of the *M. hirsuta* **stems**. B) UV spectrum. C) MS^1^ spectrum. D) MS^2^ spectrum.

A

C

B

D

**Figure S30.** A) *2′-O-trans*-coumaroylmangiferin (compound 15, RT 15.3 min), putatively identified from molecular ion *m/z* 569.1266 [M + H]^+^ in the ethanolic extract of the *M. hirsuta* **stems**. B) UV spectrum. C) MS^1^ spectrum. D) MS^2^ spectrum.

C

B

A

D

**Figure S31.** A) *2′-O-trans*-caffeoylmangiferin (compound 16, RT 14.5 min), putatively identified from molecular ion *m/z* 585.1208 [M + H]^+^ in the ethanolic extract of the *M. hirsuta* **stems**. B) UV spectrum. C) MS^1^ spectrum. D) MS^2^ spectrum.

A

B

D

C

**Figure S32.** A) Verbascoside (compound 17, RT 14.2 min), putatively identified from molecular ion *m/z* 625.2103 [M + H]^+^ in the ethanolic extract of the *M. hirsuta* **stems**. B) UV spectrum. C) MS^1^ spectrum. D) MS^2^ spectrum.

B

A

C

D

**Figure S33.** A) Isoverbascoside (compound 18, RT 14.7 min), putatively identified from molecular ion *m/z* 625.2101 [M + H]^+^ in the ethanolic extract of the *M. hirsuta* **stems**. B) UV spectrum. C) MS^1^ spectrum. D) MS^2^ spectrum.

A

B

C

D
